# SCIFER: approach for analysis of LINE-1 mRNA expression in single cells at a single locus resolution

**DOI:** 10.1186/s13100-022-00276-0

**Published:** 2022-08-26

**Authors:** Emily C. Stow, Melody Baddoo, Alexis J. LaRosa, Dawn LaCoste, Prescott Deininger, Victoria Belancio

**Affiliations:** 1 Tulane Cancer Center, Tulane Health Sciences Center, 1700 Tulane Ave, New Orleans, LA 70112 USA; 2 Department of Structural and Cellular Biology, Tulane School of Medicine, 1430 Tulane Ave, New Orleans, 70112 USA; 3 Department of Epidemiology, Tulane School of Public Health and Tropical Medicine, New Orleans, LA 70112 USA

**Keywords:** LINE1, Mobile element, Retroelement, LTR, Single cell, RNA sequencing, Expression, Testis

## Abstract

**Background:**

Endogenous expression of L1 mRNA is the first step in an L1-initiated mutagenesis event. However, the contribution of individual cell types to patterns of organ-specific L1 mRNA expression remains poorly understood, especially at single-locus resolution. We introduce a method to quantify expression of mobile elements at the single-locus resolution in scRNA-Seq datasets called **S**ingle **C**ell **I**mplementation to **F**ind **E**xpressed **R**etrotransposons (SCIFER). SCIFER aligns scRNA-Seq reads uniquely to the genome and extracts alignments from single cells by cell-specific barcodes. In contrast to the alignment performed using default parameters, this alignment strategy increases accuracy of L1 locus identification by retaining only reads that are uniquely mapped to individual L1 loci. L1 loci expressed in single cells are unambiguously identified using a list of L1 loci manually validated to be expressed in bulk RNA-Seq datasets generated from the same cell line or organ.

**Results:**

Validation of SCIFER using MCF7 cells determined technical parameters needed for optimal detection of L1 expression in single cells. We show that unsupervised analysis of L1 expression in single cells exponentially inflates both the levels of L1 expression and the number of expressed L1 loci. Application of SCIFER to analysis of scRNA-Seq datasets generated from mouse and human testes identified that mouse Round Spermatids and human Spermatogonia, Spermatocytes, and Round Spermatids express the highest levels of L1 mRNA. Our analysis also determined that similar to mice, human testes from unrelated individuals share as much as 80% of expressed L1 loci. Additionally, SCIFER determined that individual mouse cells co-express different L1 sub-families and different families of transposable elements, experimentally validating their co-existence in the same cell.

**Conclusions:**

SCIFER detects mRNA expression of individual L1 loci in single cells. It is compatible with scRNA-Seq datasets prepared using traditional sequencing methods. Validated using a human cancer cell line, SCIFER analysis of mouse and human testes identified key cell types supporting L1 expression in these species. This will further our understanding of differences and similarities in endogenous L1 mRNA expression patterns in mice and humans.

**Supplementary Information:**

The online version contains supplementary material available at 10.1186/s13100-022-00276-0.

## Background

Expression of **L**ong **IN**terspersed **E**lement-1 (LINE-1 or L1) mRNA has multiple negative consequences for genome stability. L1 mRNA expression can lead to retrotransposition of L1 transcripts or trans-retrotransposition of transcripts from other mobile elements, such as Alu or SVA [1–4]. Additionally, ORF2 and its truncated versions containing the endonuclease domain can cause DNA damage through double-strand break formation [5–7]. Experimental evidence supports that expression of retrotranspositionally incompetent L1 loci may positively or negatively affect retrotransposition of functional L1s [8, 9]. Previous studies have identified organ-, sex-, age-, and cell type-specific patterns of L1 mRNA expression using mRNA from normal or tumor samples [10–14], but many questions regarding individual L1 locus expression in different cell types remain unanswered.

L1 sequences are abundant in the human genome, which contains 500,000 truncated and full-length copies, as well as in the mouse genome, which harbors 600,000 truncated and full-length copies [15–17]. A critical concern while measuring the abundance of L1 mRNA transcripts is parsing full-length L1 mRNA transcripts produced from the L1 promoter and truncated or full-length L1 sequences incorporated into cellular transcripts via passive transcription from non-L1 promoters [13, 16, 18–22]. Detection of full-length L1 mRNA is further complicated by chimeric transcripts generated via L1 splice sites and polyadenylation signals [23–26]. Because L1 transcripts produced as a part of other genes’ transcripts are incapable of retrotransposition, they must be discarded to obtain an accurate quantification of full-length L1 mRNA transcripts [10, 12, 13, 18, 20, 27–29].

We developed a method to rigorously analyze L1 mRNA expression at a single-locus resolution by filtering out truncated and passively transcribed L1 sequences [12, 20, 30]. This method includes cytoplasmic RNA extraction, selection of polyadenylated transcripts, stranded paired-end sequencing, unique alignment of transcripts to the reference genome, and visual validation of L1 transcript alignment to annotated, full-length L1 loci [12, 20, 29, 30]. All existing methods developed for single-locus L1 expression [13, 28], including our own [12, 20, 30], have limitations in their ability to reproducibly detect all L1 subfamilies in datasets with variable sequencing depth. Our method is specifically limited in its ability to detect expression from most L1Hs loci, the least diverged L1 subfamily, due to our alignment parameters which require unique sequencing read alignments [12, 20, 30]. Despite this limitation it is a useful tool to discover expression patterns of unambiguously expressed L1 loci. Previous application of this approach demonstrated organ-, age, and sex-specific L1 loci expression as well as identified epigenetic features of expressed L1 loci [10, 29]. The last frontier in L1 expression remains rigorous detection of individual L1 or other transposable elements expression in single cells. In contrast to cellular genes, conventional technical approaches and bioinformatics pipelines are not suitable for detection of repetitive sequences [10, 20, 21]. For example, traditional single cell RNA sequencing (scRNA-Seq) approaches using 10X Chromium Single Cell 3′ Genomics Technology target the polyadenylated end of transcripts for sequencing [31]. Although this method is efficient for accurate detection of expression from single genes, loss of information regarding the rest of the transcript makes it impossible to separate full-length from truncated L1 transcripts produced by passive transcription with scRNA-Seq alone. Additionally, 10X Genomics Software does not have an option to conduct an exhaustive and unique alignment, a necessary step for assigning repetitive L1 transcripts to their correct locus of origin [12, 20, 30]. The 10X Chromium Single Cell 3′ Genomics approach has been used to generate large publicly available scRNA-Seq datasets, which could be informative for expression patterns of repetitive elements if an appropriate method was available for their analysis.

Methods to quantify transposable element expression in single cells, such as the study by Shao, et al,. [32] implement the use of bulk RNA-Seq generated transcripts to reduce noise in scRNA-Seq analysis but retain multi-mapping reads. This approach likely inflates the number of expressed L1 loci and reduces the ability to identify and accurately quantify L1 expression at the locus-specific level [32]. scTE introduced by He, et al. quantifies TE expression by subfamily, allowing the retention of multimapped reads based on assignment to the highest scoring locus, and is therefore unable to identify L1 expression at the locus-specific level [33]. CELLO-Seq, a method introduced by Berrens, et al., uses a combination of single cell long-read sequencing and short read sequencing to assign reads uniquely to L1 loci and quantify locus-specific expression [34]. While CELLO-Seq includes the appropriate steps to accurately measure L1 expression, it is costly and cannot be applied to the large number of 10X Chromium Single Cell 3′ generated scRNA-Seq datasets that are publicly available [35–42]. Thus, development of custom approaches for bioinformatics analysis of L1 loci expression in single cells that work with 10X Chromium Single Cell 3′ Genomics datasets and is compatible with other sequencing platforms will increase our ability to gain information about cell types potentially vulnerable to downstream effects of L1 expression.

Here, we report a new bioinformatics tool designed for analysis of L1 mRNA expression quantification from individual L1 loci in single cells called **S**ingle **C**ell **I**mplementation for **F**inding **E**xpressed **R**etrotransposons (SCIFER). SCIFER extracts scRNA-Seq reads generated from traditional 10X Chromium Single Cell 3′ Genomics sequencing along with their barcodes and realigns them to the reference genome using Bowtie [43] with unique (−m1) and tryhard (−y) settings, the same Bowtie settings used in our previously reported method [10, 12, 20, 30]. While this approach unambiguously identifies expressed L1 loci, it significantly reduces sensitivity of detection of L1Hs loci, which are the least diverged L1 subfamily. SCIFER also validates authentic L1 expression by comparing scRNA-Seq data with a list of full-length L1 loci determined to be expressed in bulk RNA-Seq analysis using our unique alignment settings and visual validation of each L1 locus [12, 20, 30]. SCIFER analysis of L1 mRNA expression in MCF7 cells determined the required sequencing coverage of scRNA-Seq for reliable detection of L1 expression and identified other parameters that influence L1 detection, such as the L1 locus expression level in bulk RNA-Seq and the number of cells expressing the L1 locus. SCIFER analysis of mouse testis scRNA-Seq identified that Round Spermatids express the highest levels of L1 mRNA and number of L1 loci per cell. Analysis of L1 mRNA expression in human testes using bulk RNA-Seq identified that human testes from unrelated individuals, similar to mouse testes, share over 80% of expressed L1 loci. Additionally, SCIFER analysis identified that a significant majority of L1 mRNA expression occurs in similar cell types in mouse and human testes with Spermatogonia, Spermatocytes, and Round Spermatids expressing the highest L1 mRNA levels in human testes.

## Results

### SCIFER workflow

**Fig. 1:**
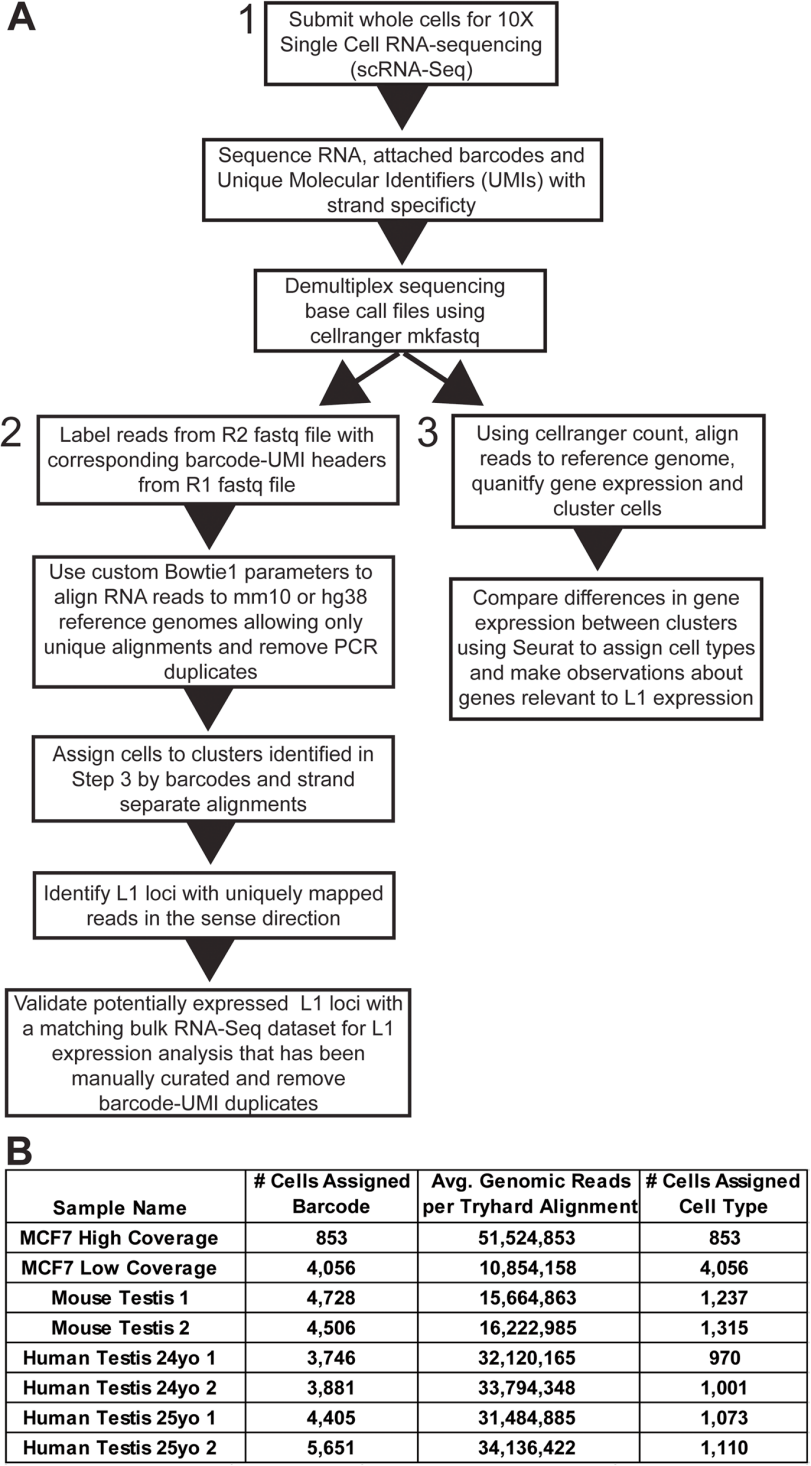
**S**ingle **C**ell **I**mplementation to **F**ind **E**xpression of **R**etrotransposons (SCIFER) incorporates information from visually validated bulk RNA-Seq to accurately measure L1 mRNA expression in scRNA-Seq datasets. **A**. The SCIFER method workflow is shown. Single cells are sequenced and demultiplexed using 10X Genomics cellranger tools (1). Reads are labeled with their corresponding barcode, reads are aligned uniquely to the reference genome, PCR duplicates are removed by alignment and UMI, alignments are strand separated, and alignments are compared to a list of authentically expressed L1 loci from a bulk RNA-Seq dataset in a matched sample (2). Reads are aligned using 10x Genomics and gene expression is quantified using Seurat (3). **B**. A table showing the number of cells captured per dataset, the average number of genomic aligned reads using the tryhard bowtie settings per cell (see Methods), and the number of cells with an assigned cell type per dataset. Values are listed for datasets: MCF7 High coverage, MCF7 Low coverage [44], Mouse Testis 1 and 2 [37], and Human Testis 24 yo and 25 yo donors [35]

SCIFER (**S**ingle **C**ell **I**mplementation to **F**ind **E**xpressed **R**etrotransposons) is a method to quantify L1 mRNA expression at the locus-specific level in scRNA-Seq datasets. SCIFER aligns data from a standard 10X Chromium Single Cell 3′ RNA-Seq dataset to the reference genome using procedures optimized to deal with the very high genomic copy number of L1 elements (Fig. 1A, see Methods). 10X Chromium Single Cell 3′ RNA-Seq read alignments are enriched at the 3′ end of L1 loci and genes due to the selection of polyadenylated transcripts during library preparation. Therefore, scRNA-Seq data lack equal distribution of read alignments across the expressed L1 locus, preventing the analysis of 5′ aligned reads that allow discernment between authentically and passively transcribed L1 loci [12, 20, 30]. For this reason, SCIFER requires that bulk RNA-Seq analysis of L1 expression using a list of full-length L1 loci has been performed on a matching sample, allowing for validation of L1 loci that were exclusively expressed from the L1 promoter. Additionally, SCIFER performs an alignment to the reference genome, instead of the transcriptome, to allow detection of L1 elements in introns and intergenic regions (Fig. 1, step 2).

To validate SCIFER efficacy we performed scRNA-Seq of MCF7 and HEK293-FRT-LacZeo cells in a combined sample using the 10X Genomics Chromium™ Single Cell 3′ Library & Gel Bead Kit v3’. To assess bias in the representation of scRNA-Seq reads, bulk RNA-Seq reads and genomic DNA-Seq reads from MCF7 cells and scRNA-Seq reads from our sample were separately aligned to the L1 consensus sequence (Additional file 1 A). DNA-seq and bulk RNA-Seq reads were distributed throughout the L1 sequence with some enrichment at the 3′ end due to the abundance of 5′ truncated L1 loci in the human genome. Alignment of scRNA-Seq reads to the 3′ end of the L1 consensus were 2X more abundant than alignments to other regions of the L1 sequence (Additional file 1 A), reflective of the 3′ targeted sequencing procedure. Similar analysis of actin (ACTB) demonstrated the expected enrichment of reads at the 3′ end of the gene locus in scRNA-Seq, with bulk RNA-Seq reads evenly distributed throughout gene exons (Additional file 1 B, top). Bulk RNA-Seq reads are equally distributed throughout the length of an L1 locus previously identified and authenticated as expressed (Additional file 1 B, bottom) [29]. Alignment of scRNA-Seq reads to the same L1 locus are enriched at the 3′ end (Additional file 1 B, bottom). Based on these comparisons, the use of the traditional 3′ targeted scRNA-Seq alone is not an adequate method to detect L1 expression (Additional file 1 B). Therefore, we used expression data from bulk RNA-seq to accurately detect expressed L1 loci to guide SCIFER analysis.

### Analysis of L1 mRNA expression in MCF7 cells

**Fig. 2:**
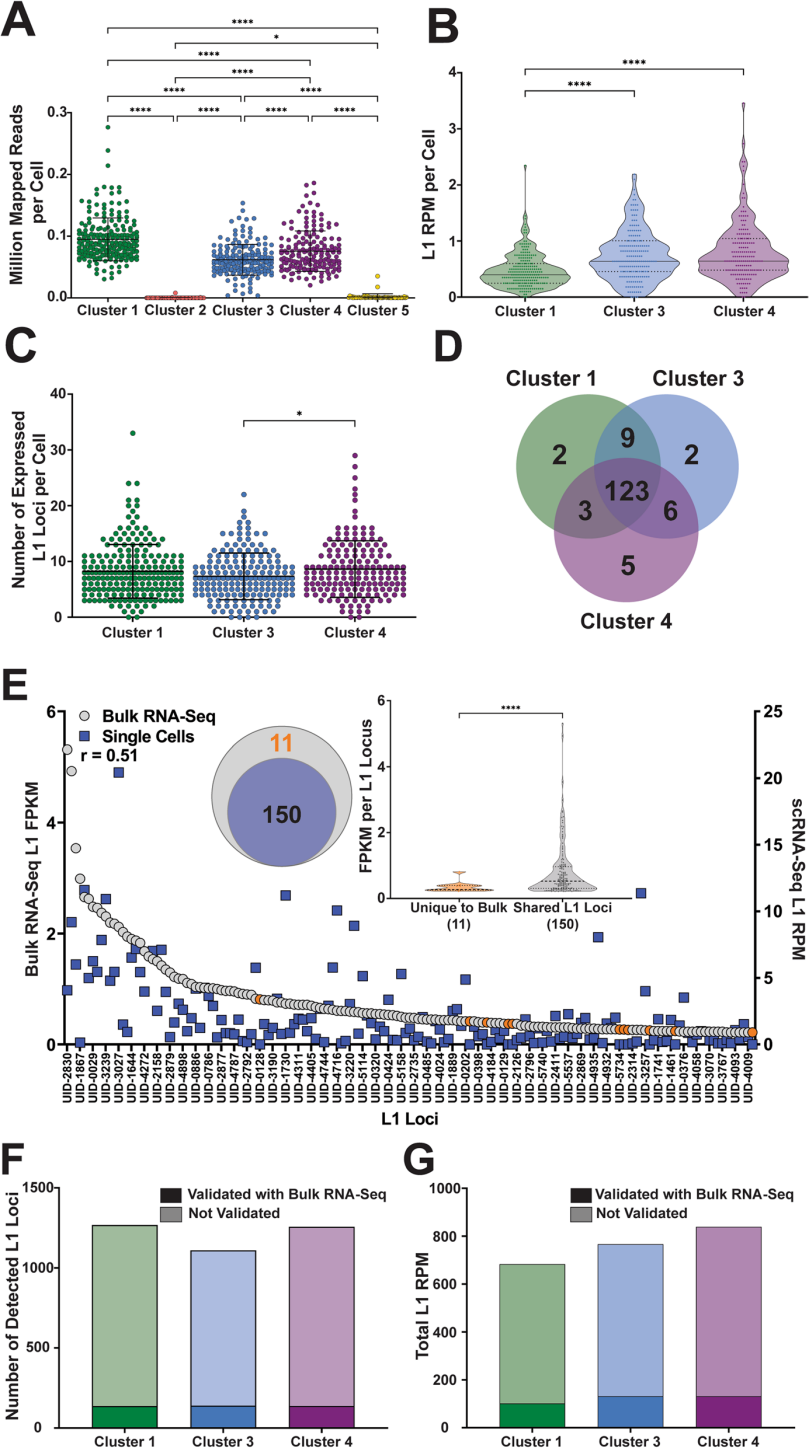
L1 mRNA expression is consistent in MCF7 single cell clusters. **A**. The number of million mapped reads per cell is shown for each tSNE cluster of MCF7 cells (ANOVA, ****,< 0.0001). **B**. The L1 expression level per cell quantified by RPM is shown for Clusters 1, 3, and 4 in the Violin Plot (ANOVA, ****,< 0.0001). **C**. The number of expressed L1 loci per cell is shown for MCF7 clusters 1, 3, and 4 (ANOVA, *, < 0.05). **D**. The number of expressed L1 loci shared between MCF7 clusters 1, 3, and 4 is shown in the Venn diagram. **E**. The L1 FPKM values for bulk RNA-Seq (left y-axis) and L1 RPM values for High coverage scRNA-Seq (right y-axis) of MCF7 cells are shown in the dot-plot. Orange circles indicate L1 loci with expression detected in the bulk dataset that were not detected to be expressed in the scRNA-Seq dataset. The nested Venn diagram shows the number of shared expressed L1 loci from the bulk and scRNA-Seq datasets. The violin plot shows the FPKM values for expressed L1 loci unique to the bulk dataset and those shared between bulk and scRNA-Seq (Welch’s *t*-test, ****,< 0.0001). **F**. The number of expressed L1 loci validated with bulk RNA-Seq is shown in darker colors with the number of expressed L1 loci without bulk RNA-Seq confirmation shown in lighter colors. **G**. The L1 RPM levels per MCF7 cluster for L1 loci confirmed with bulk RNA-Seq are shown in dark colors with the RPM levels for L1 loci with no bulk RNA-Seq confirmation shown in light colors

To validate the utility of SCIFER for detection of L1 expression in scRNA-Seq datasets and to identify technical strengths and limitations of this approach, we analyzed High and Low coverage MCF7 scRNA-Seq datasets (Fig. 1B). We performed High coverage scRNA-Seq on a pool of MCF7 and HEK293-FRT-LacZeo cells in a 9:1 ratio, respectively, using a total of 853 cells in this experiment (Fig. 1B). Clustering by 10X Genomics cellranger count identified that MCF7 cells formed 5 clusters (Additional file 2 A) with Cluster 1 cells having on average significantly higher numbers of mapped reads per cell compared to Clusters 3 and 4 (*P* < 0.0001, Fig. 2A). Clusters 2 and 5 contained low numbers of RNA molecules and Cluster 2 contained a high percentage of mitochondrial read;, therefore, both were discarded from further analysis (Fig. 2A, Additional file 2 B). HEK293-FRT-LacZeo cells were used as an internal control within our cell mixture for accurate cell clustering. After sequencing and clustering cells using the cellranger count tool, the HEK293-FRT-LacZeo cells clustered separately from the MCF7 cells (Additional file 2 A, Clusters 6 and 7). HEK293-FRT-LacZeo cell identity was confirmed by aligning reads to the FRT-LacZeo transgene sequence.

Following alignment, clustering of cells, and de-duplication of barcode-UMIs, the next step in SCIFER analysis is to parse expressed L1 loci from passively transcribed L1 sequences (also referred to as background) by cross referencing the list of L1 loci that were assigned same-sense RNA-Seq alignments in the scRNA-Seq dataset with a list of full-length L1 loci validated to be expressed in MCF7 cells from a previous study (Additional file 17, Fig. 1, step 2) [29]. By summing the RPM of all L1 loci identified as expressed by this approach in each MCF7 cell, we determined the RPM levels per cell for each cluster (Fig. 2B). The three MCF7 clusters with considerable L1 expression (Clusters 1, 3, and 4) had similar average L1 expression levels ranging from 0.48 RPM in Cluster 1 to 0.80 RPM in Cluster 4 (Fig. 2B). Differences in L1 expression levels between clusters 1, 3, and 4 were also compared using Seurat v4.0.5 (Additional file 2 E). MCF7 clusters 1, 3, and 4 also expressed similar numbers of L1 loci with the average number of expressed L1 loci per cell ranging from 7.3 in Cluster 3 to 8.6 in Cluster 4 (Fig. 2C). Comparison of the L1 loci expressed in Clusters 1, 3, and 4, determined that the clusters share 82% (123 out of 150) of expressed L1 loci (Fig. 2D). The L1 RPM and number of expressed L1 loci for Clusters 2 and 5 were excluded from these figures due to not meeting technical standards, but comparisons of L1 RPM and the number of expressed L1 loci that include these clusters are shown in Additional file 2 C and D.

To find how closely SCIFER analysis of scRNA-Seq mirrors bulk RNA-Seq detection of L1 expression using our validation method [20, 30], we compared the list of L1 loci manually validated to be expressed in bulk RNA-Seq with the list of L1 loci that received ≥1 sequence alignment in scRNA-Seq. 150 of the 161 L1 loci (93%) identified as expressed in bulk RNA-Seq were detected to be expressed in scRNA-Seq (Fig. 2E, Venn diagram). We observed that the RPM per L1 locus detected using scRNA-Seq positively correlates with L1 FPKM determined using bulk RNA-Seq (*r* = 0.51, *P* < 0.0001, Fig. 2E). Comparing the bulk RNA-Seq FPKM level of the 11 L1 loci that were not detected to be expressed by scRNA-Seq revealed that the L1 loci unique to the bulk RNA-Seq dataset were expressed at a significantly lower FPKM compared to the L1 loci detected by both bulk and Single Cell RNA-Seq (*P <* 0.0001, Fig. 2E, Violin plot). The positive correlation of L1 FPKM detected in bulk RNA-seq with L1 RPM observed in scRNA-Seq dataset demonstrates that SCIFER performs accurate detection of L1 expression at the locus-specific level in single cells. The ability of SCIFER to detect 93% of the L1 loci determined to be expressed in the bulk RNA-Seq analysis also demonstrates that this approach is sensitive enough to identify almost all expressed L1 loci, missing L1 loci that have, on average, significantly lower expression levels than the loci detected to be expressed (Fig. 2E, Violin plot).

Several published studies employ analysis of scRNA-Seq reads to draw conclusions regarding TE expression in single cells without considering limitations of this technology such as the presence of passive L1 expression that requires careful analysis of potentially expressed L1 loci to address [32, 33]. To determine the outcome of unsupervised analysis of L1 expression using 10X Chromium Single Cell 3′ scRNA-Seq methodology, all L1 loci with unique alignments identified in scRNA-Seq were considered as potentially expressed. Comparison of unsupervised with bulk-validated L1 loci determined that the number of expressed L1 loci per cluster was inflated 8-9X and the total L1 RPM per cluster was inflated 5-6X per cluster in the non-validated dataset (Fig. 2F and G). This discrepancy highlights the importance of including a list of expressed, full-length L1 loci validated in bulk RNA-Seq analysis from a matching sample to guide findings made using scRNA-Seq datasets. Without such guidance, scRNA-Seq analysis of TE expression produces results that lack scientific rigor and biological meaning by disproportionally exaggerating both the levels of L1 expression and the number of expressed L1 loci.

### Shallow sequencing reduces sensitivity of L1 mRNA expression detection in single cells

Sequencing depth is an important consideration when preparing samples for analysis of mobile element expression due to their low expression level in normal tissues, especially at the locus-specific level [10, 20, 21, 45–47]. Because most publicly available scRNA-Seq datasets are not sequenced to as high of a depth as our High coverage MCF7 dataset (Fig. 1B), we next tested the effect of reduced sequencing coverage on SCIFER’s ability to detect expressed L1 loci and their expression levels in single cells. To accomplish this, we used two complementary approaches: downsampling of high coverage cells from the MCF7 High coverage dataset and analysis of an independent MCF7 Low coverage scRNA-Seq dataset.

**Fig. 3:**
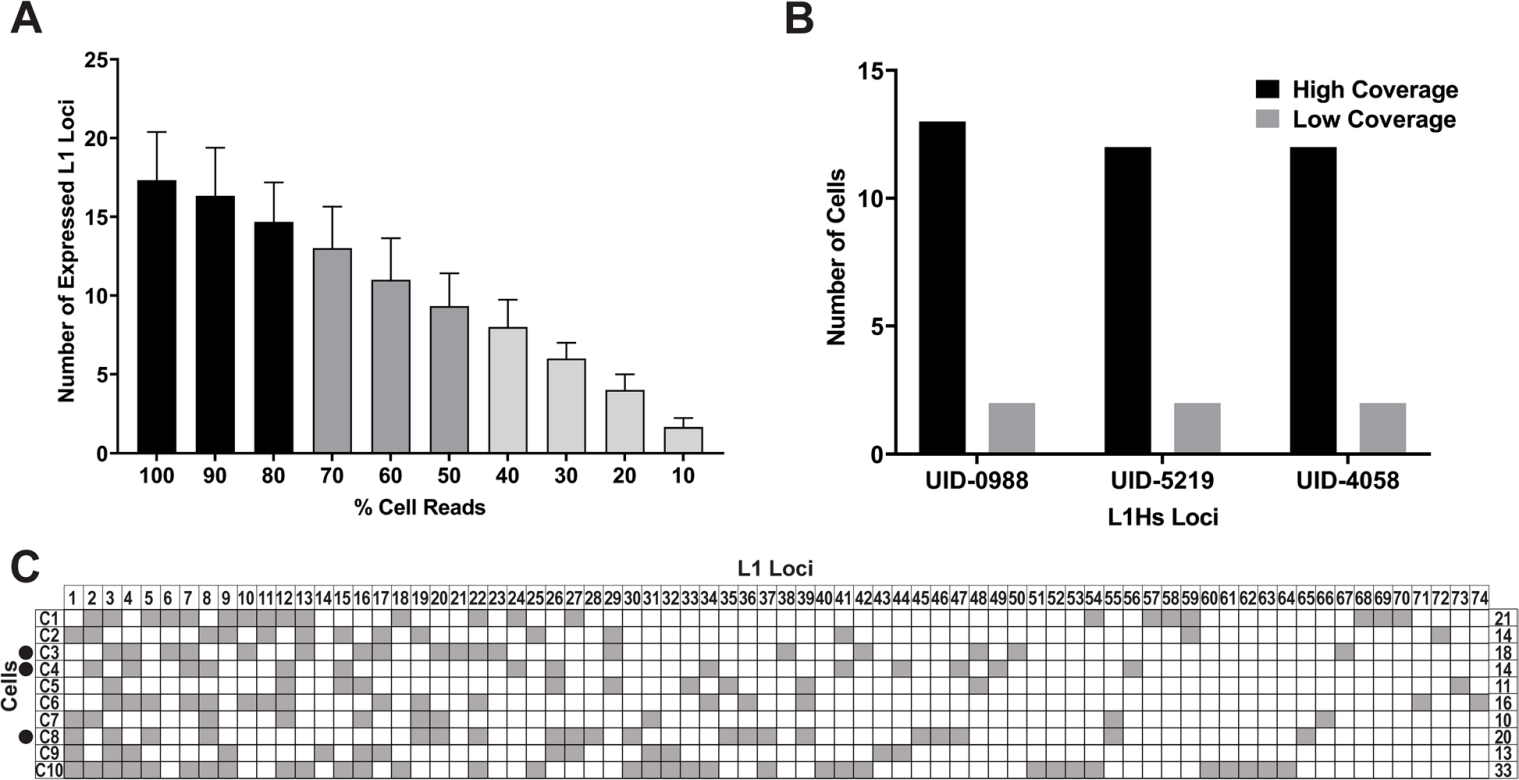
Detection of L1 mRNA expression in MCF7 single cells is sequencing depth dependent. **A**. The average number of expressed L1 loci detected in 3 MCF7 cells is shown with decreasing read depth (100–10% of total cell reads). The black bars have significantly higher numbers of detected L1 loci compared to the gray bars (Welch’s *t*-test, 100% vs. 70%, *P =* 0.019). The lightest gray bars (30–10%) indicate detection of less than half of expressed L1 loci detected with 100% of reads. **B**. The number of cells expressing L1Hs loci is shown for High and Low coverage MCF7 scRNA-Seq. **C**. A correlation matrix of L1 loci expressed in 10 MCF7 cells. The L1 loci are listed by number at the top of the matrix and the 10 cells are listed along the left side. Filled boxes indicate that the L1 was expressed in the corresponding cell. Black dots indicate the cells used in (**A**)

Three MCF7 cells with similar sequencing depth (0.17–0.18 million mapped reads per cell) were down sampled in 10% intervals and the average number of expressed L1 loci detected in each resulting interval sample was compared (Fig. 3A). This approach demonstrated that the average number of L1 loci expressed in these three cells drops off significantly at 70% of the original sample size (ANOVA, *P =* 0.026, Fig. 3A). This established that optimum detection of expressed L1 loci by SCIFER occurs at ≥80% of our starting reads (≥0.14 million mapped reads per cell) and at least half of expressed L1 loci remain detectable in files containing 50% of the original number of reads (Fig. 3A).

To determine limitations imposed by lower sequencing coverage, we performed SCIFER analysis on a low coverage MCF7 scRNA-Seq dataset with 10-fold less sequencing coverage and 5-fold more sequenced cells compared to the High coverage MCF7 scRNA-Seq dataset (Fig. 1B, Additional file 2 B). The cluster with the highest average number of mapped reads in this dataset (Cluster 7, 0.0032 million mapped reads per cell) had on average ~ 30X fewer mapped reads than Cluster 1 from the High coverage dataset, the cluster with the highest average number (0.095) of million mapped reads in the High coverage dataset (*P <* 0.0001, comparing Fig. 2A Cluster 1 and Additional file 3 A Cluster 7). The Low coverage dataset also had ~29X fewer expressed L1 loci per cell compared to the High coverage dataset (0.3 vs. Cluster 4 High: 8.6 expressed L1 loci) and the L1 RPM was ~ 1.9X lower in the Low than the High coverage dataset (0.35 vs. 0.66 L1 RPM, Fig. 2B and C and Additional file 3 B and C). Bulk RNA-Seq of MCF7 cells followed by manual validation identified three L1Hs loci as expressed [12, 20, 29, 30]. Because L1Hs loci are the evolutionarily youngest L1s in the human genome and, therefore the hardest to map uniquely by all approaches, including ours, but the most capable of contributing to L1-related genome instability, we considered whether their detection would change with sequencing depth. We quantified the number of cells expressing each of the three L1Hs loci in a High and Low coverage scRNA-Seq dataset of MCF7 cells (Fig. 1B, Fig. 3B). We found that while all three L1Hs loci were detected in both High and Low coverage scRNA-Seq, the L1Hs loci expression was detected in 6.2X fewer cells in the Low coverage dataset compared to the High-coverage, on average (Fig. 3B). These findings demonstrate that accurate detection of L1Hs expressing cells is reduced in lower coverage scRNA-Seq datasets leading to an underestimation of L1 expression and its potential biological impact.

We next compared the total L1 expression levels detected by SCIFER in the Low coverage MCF7 dataset with L1 expression levels detected in the bulk RNA-seq dataset. Despite reduced sensitivity of detection of L1 expression per cell in the Low coverage dataset, RPM per expressed L1 locus in the Low coverage dataset was positively correlated with bulk RNA-Seq L1 FPKM (*r* = 0.55, *P* < 0.0001 Additional file 3 E). Additionally, 88% (142 of 161) of L1 loci shown to be expressed in bulk are detected as expressed in the Low coverage MCF7 dataset (Additional file 3 E, Venn diagram). The L1 loci unique to bulk RNA-Seq had significantly lower FPKM levels compared to the FPKM levels of the L1 loci shared between bulk and scRNA-Seq (Additional file 3 E, Violin Plot, *P <* 0.0001). The positive L1 FPKM-RPM correlation and high percent of shared expressed loci detected between Low coverage scRNA-Seq and bulk RNA-Seq establishes that L1 expression detection, averaged across a population of cells, is not dramatically impacted by reduced sequencing depth. However, the number of expressed L1 loci per cell and their levels of expression are underestimated when a low coverage dataset is used. Additionally, similar to the High coverage scRNA-Seq dataset, L1 loci expressed in bulk RNA-Seq but missed by SCIFER in lower sequencing coverage scRNA-Seq datasets are those L1 loci that have low expression levels.

Guided by these findings we analyzed L1 loci expression detected by SCIFER in 10 single MCF7 cells with similar and highest sequencing depth (0.17–0.28 million mapped reads per cell) to determine the extent of variation in L1 expression between individual cells (Fig. 3C). This analysis demonstrated that some L1 loci were expressed by 6–7 cells, such as loci L1–3027, L1–4594 and L1–4591 (loci 3, 8,and 12, respectively), while others were expressed only in one cell, such as L1-1644, L1-3511, L1-5118, L1-0862, L1-1741, L1-4311, L1-0320, L1-2843, L1-5594, L1-2879, L1-4279, L1-0424, L1-2543, L1-2693, L1-3746, L1-4326, L1-3864, L1-4757, L1-1889, L1-4296, L1-4093, L1-4644, L1-4405, L1-4024, L1-5096, L1-4935, L1-3602 (loci 14, 21, 23, 38, 49-53, 56-58, 60-74, respectively) (Fig. 3C). The number of expressed L1 loci per cell varied from 10 in cell 7 to 33 in cell 10 (Fig. 3C). This cell-to-cell variation can be due to the drop-out of the detected L1 locus expression, an inherent feature of scRNA-Seq experiments.

We also detect expression of housekeeping genes (HKGs) in cells with read numbers downsampled in 10% increments, the same three cells used in Fig. 3A (Additional file 2 F and G). We observed that while some HKGs were more robustly expressed than others (Additional file 2 F), the average number of detected HKGs did not significantly decrease until 40% of the starting number of reads (Additional file 2 G). The difference in the dropout threshold between HKG and L1 (40% vs. 70%, respectively, Fig. 3A and Additional file 2 G) is likely due to individual L1 loci having lower levels of expression than genes tested in this experiment. We also generated an expression matrix of HKGs in the 10 high-sequencing depth cells from Fig. 3C. We observe that HKG expression detection is more uniform across single cells compared to expression of L1 loci (Additional file 2 H and Fig. 3C). Notably, HKGs with lower expression levels (Additional file 2 F) demonstrate drop-out amongst the 10 high-sequencing depth cells (Additional file 2 H). These findings show that the depth of sequencing is an important consideration when investigating L1 expression patterns or expression patterns of genes with low expression levels. This analysis also supports that biologically informative observations about single L1 locus expression or genes with low expression levels should be made at the cell cluster or cell type level, rather than between individual cells. With this in mind, given the high sequence depth of the 10 cells analyzed in this experiment, our findings of cell-to-cell variation in locus-specific L1 expression could partially reflect biologically relevant patterns of L1 expression in individual cells, which would be consistent with previously observed differences in L1 retrotransposition events between single cells [48].

### L1 mRNA expression in single cells of mouse testes

Previously, our comprehensive analysis of locus-specific L1 mRNA expression in mouse organs determined that testes express the highest levels of L1 mRNA compared to other mouse organs, including male and female brains, male and female lungs, ovaries, and uteri [10]. To determine the cellular source(s) of L1 expression in mouse testes, we used SCIFER to analyze scRNA-Seq data from testes collected from two 2 mo mice. First, expression of spermatogenesis-specific cell markers was used to confirm correct cell clustering in the mouse testis samples. TBPL1 is a marker of Spermatocyte and early Spermatid stages and was found to have significantly higher expression in Spermatocyte and Round Spermatid clusters compared to Elongating and Condensing Spermatids (*P <* 0.0001, Additional file 17, Additional file 4 A1, B1, and C) [49]. PRM1, a protamine that is exchanged for histones during the haploid phase of spermatogenesis, was expressed significantly higher in Round, Elongating, and Condensing Spermatids compared to Spermatocytes and Sertoli cells (*P <* 0.0001, Additional file 17, Additional file 4 A2, B2, and C) [49]. TNP1, a protein involved in the histone-protamine exchange, also had significantly higher expression in Round, Elongating, and Condensing Spermatids compared to Spermatocytes and Sertoli cells (*P <* 0.0001, Additional file 17, Additional file 4 A3, B3, and C) [49]. These gene expression profiles are consistent with accurate clustering of mouse testis cells prior to SCIFER analysis.

**Fig. 4:**
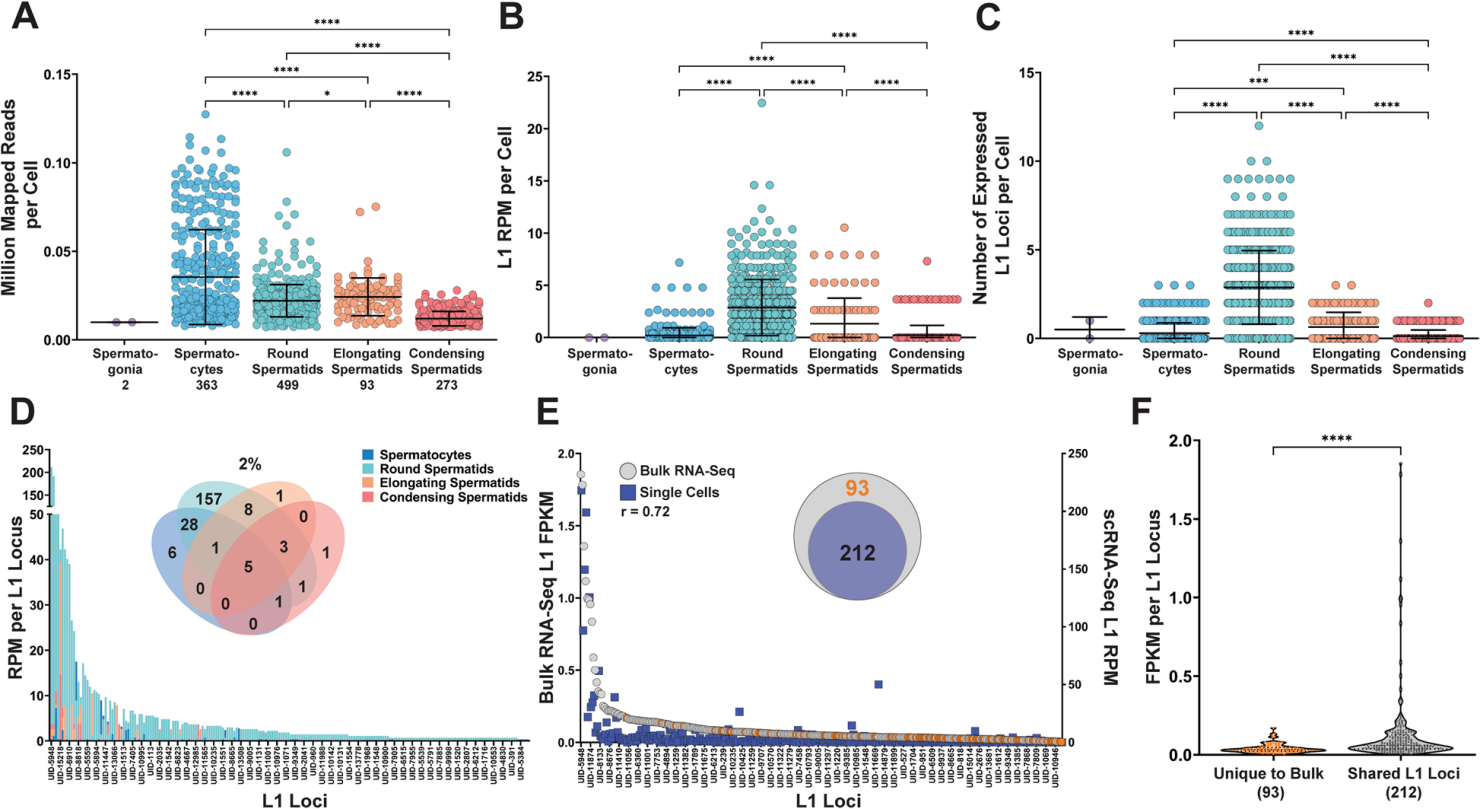
SCIFER detects cell type specific patterns of L1 mRNA expression in mouse testes. **A**. The number of million mapped reads per cell for each cell type is shown in the individual value plot for Mouse 1 (ANOVA, ****,< 0.0001, *, < 0.05). **B**. L1 mRNA expression quantified by RPM for each cell is shown in the individual value plot for Mouse 1 (ANOVA, ****,< 0.0001). **C.** The number of expressed L1 loci per cell in each cluster is shown in the individual value plot for Mouse 1 (ANOVA, ***, < 0.0005, ****,< 0.0001). **D**. The L1 RPM values for L1 loci indicated on the X-axis are shown for each sperm cell type in Mouse 1. The Venn Diagram shows the number of expressed L1 loci shared by the sperm cell types with the percentage of shared expressed L1 loci indicated above the diagram. **E**. The L1 FPKM values for bulk RNA-Seq (left y-axis) and L1 RPM values for scRNA-Seq (right y-axis) of mouse testis are shown in the dot-plot. Orange circles indicate L1 loci with detected expression in the bulk dataset that were not detected to be expressed in scRNA-Seq. The nested Venn diagram shows the number of shared expressed L1 loci from the bulk and scRNA-Seq datasets. **F**. The violin plot shows the FPKM values for expressed L1 loci unique to the bulk dataset and those shared between bulk and scRNA-Seq (Welch’s *t*-test, ****,< 0.0001)

SCIFER analysis was performed on the scRNA-Seq mouse testis samples to discover the levels and patterns of L1 mRNA expression in different cell types using a list of L1 loci validated to be expressed in mouse testes from a previous publication [10]. SCIFER analysis of the scRNA-Seq mouse testis datasets found that Round Spermatids express on average the highest levels of L1 per cell compared to other cell types (Fig. 4B and C). They have higher average L1 RPM per cell, compared to clusters of Spermatocytes, Elongating Spermatids, and Condensing Spermatids (ANOVA, *P <* 0.0001, *P <* 0.0001, *P <* 0.0001, respectively, Fig. 4B). Analysis of L1 RPM with Seurat v4.0.5 analysis confirmed that Round Spermatids had on average significantly higher L1 expression levels compared to Spermatocytes, Elongating Spermatids, and Condensing Spermatids (Wilcoxon rank sum, *P* = 4.39E-99, *P* = 1.20E-28, *P* = 3.89E-94, respectively, Additional file 4 D). Mouse Round Spermatids also on average express more L1 loci per cell (2.9 loci) than other cell types with some cells expressing 8–12 L1 loci (compared to Spermatocytes, Elongating Spermatids, and Condensing Spermatids which, on average, express less than one locus per cell (ANOVA, *P <* 0.0001, *P <* 0.0001, *P <* 0.0001, respectively, Fig. 4C). Too few Spermatogonia, Leydig, and Sertoli cells were identified in these datasets to make meaningful comparisons (Additional file 5 C and E). SCIFER was used to analyze a second mouse dataset of a lower sequencing coverage (Fig. 1B, Additional file 5 B) and confirmed that on average Round Spermatids in Mouse 2 also expressed L1 at a significantly higher RPM (Additional file 5 D) and on average expressed a significantly higher number of L1 loci per cell compared to the other cell types (Additional file 5 F). These findings establish that Round Spermatids reproducibly express the highest levels of L1 mRNA compared to other cell types in mouse testes.

To understand potential changes in L1 expression during spermatogenesis, we compared the identity of L1 loci expressed in different sperm cell types identified in our single cell pool. Comparison of L1 loci expressed in different sperm cell types determined that in the Mouse 1 dataset, Spermatocytes, Round Spermatids, Elongating Spermatids, and Condensing Spermatids share only 2% (5 of 212) of L1 loci expressed in testes, with 157 L1 loci being detected to be expressed only in Round Spermatids alone (Fig. 4D). In the Mouse 2 dataset, 0% (0 of 217) of expressed L1 loci are shared by Spermatocytes, Round Spermatids, Elongating Spermatids, and Condensing Spermatids with Round Spermatids expressing 160 unique L1 loci (Additional file 5 G). The high number of expressed L1 loci unique to Round Spermatids in both mouse replicates (157 and 160) demonstrates that Round Spermatids reproducibly support the majority of L1 loci detected to be expressed in bulk RNA-Seq analysis even though their genome is haploid. We also observe a high level of similarity in the expressed L1 loci shared between Mouse 1 and Mouse 2 Round Spermatids (65%, 162 of 251, Additional file 5 H2). In comparison, the number of shared loci between Mouse 1 and 2 is 31% in Spermatocytes, 44% in Elongating Spermatocytes, and 0% in Condensing Spermatids (Additional file 5 H1, H3, and H4). Additionally, Round Spermatids are the most abundant cell type in both Mouse 1 and Mouse 2 samples representing 40% (499 of 1237, including Leydig and Sertoli cells) and 49% (641 of 1315, including Leydig and Sertoli cells) of the cell populations, respectively, which may lead to their L1 expression levels dominating the SCIFER analysis. Of note, Spermatocytes are the second most abundant cell type in both Mouse 1 and Mouse 2 representing 30% (363 of 1237) and 25% (330 of 1315) of the cell populations, respectively, yet Spermatocytes have significantly lower L1 RPM levels and fewer expressed L1 loci (Fig. 4B and C, Additional file 5 D and F). This suggests that the high levels of L1 expression observed in Round Spermatids are biologically relevant and not a reflection of oversampling of the cell type.

Previously we found that in MCF7 cells SCIFER detects 88–93% of expressed L1 loci detected in bulk RNA-Seq and the L1 FPKM-RPM levels of expressed L1s are positively correlated between SCIFER-detected L1 loci expression in single cells and bulk RNA-Seq (*r* = 0.51, *P* < 0.0001, Fig. 2E and Additional file 3 E). To determine whether SCIFER is similarly consistent with bulk RNA-Seq L1 mRNA detection in an organ-derived sample with multiple cell types, we assessed the expression levels of L1 loci and compared the identity of expressed L1 loci in SCIFER analyzed scRNA-Seq and bulk RNA-Seq generated using 2mo mouse testes. With this analysis we found a strong positive correlation between L1 loci expression in scRNA-Seq and bulk RNA-Seq of mouse testes (*r* = 0.72, *P* < 0.0001, Fig. 4E). We also found that 212 of the 305 L1 loci identified as expressed using bulk RNA-Seq of mouse testes are identified by SCIFER analysis of scRNA-Seq of mouse testes (Fig. 4E, Venn Diagram). The FPKM levels of the 93 L1 loci detected as expressed in bulk RNA-Seq but not in scRNA-Seq were significantly lower than the 212 expressed L1 loci detected in both bulk RNA-Seq and scRNA-Seq with SCIFER (Welch’s t-test, *P <* 0.0001, Fig. 4F). These loci could also be expressed in other cell types such as Spermatogonia, Leydig, and Sertoli cell types that are underrepresented in these scRNA-Seq datasets and most likely in the bulk RNA-Seq datasets as well. Furthermore, based on our findings in MCF7 cells, an increase in sequencing depth could have resulted in a greater number of L1 loci shared between bulk and scRNA-Seq datasets.

Variable L1 mRNA expression detected by SCIFER in different cell types represented in scRNA-Seq of mouse testes led us to consider the expression of genes previously identified to contribute to restriction of L1 expression and translation during Spermatogenesis [50]. We quantified expression levels of Pld6 and Hsp90aa1, two piRNA pathway genes involved in the restriction of L1 activity in mouse testis [50], in mouse testis cell types. PLD6 is a piRNA pathway protein involved in the processing of piRNAs during Spermatogenesis [50, 51]. We observed that Pld6 is expressed at the highest level in Spermatocytes (Additional file 6 A1, B1, and C, Mouse 1: 0.91, Mouse 2: 1.04 Normalized Expression) and second highest level in Round Spermatids (Additional file 6 C, Mouse 1: 0.25, Mouse 2: 0.22 Normalized Expression), compared to the other sperm cell types (Additional file 6 A1, B1, and C). Hsp90aa1 is similarly expressed at the highest level in Spermatocytes (Additional file 6 A2, B2, C, Mouse 1: 3.01, Mouse 2: 3.12 Normalized Expression) and Round Spermatids (Additional file 6 A2, B2, C, Mouse 1: 2.24, Mouse 2: 2.21 Normalized Expression). We also considered the expression level of UHRF1, a protein that recruits DNMT1 and promotes DNA methylation at hemimethylated CpGs [50, 52, 53]. We observe decreasing levels of Uhrf1 in Round Spermatids (Additional file 6 A3, B3, C, Mouse 1: 0.29, Mouse 2:0.30) compared to Spermatocytes (Additional file 6 A3, B3, C, Mouse 1: 0.66, Mouse 2:0.67). The overall patterns of Pld6, Hsp90aa1, and Uhrf1 expression, genes related to inhibiting L1 expression and translation [50, 51, 54–57], in mouse testes are consistent with the observed peak in L1 expression that SCIFER detects in Round Spermatids (Additional file 6). This initial analysis demonstrates that the increase in L1 expression observed in Round Spermatids coincides with a peak and subsequent downregulation of Uhrf1 expression, a component of the DNA methylation pathway, as well as a peak and subsequent downregulation of Pld6 and Hsp90aa1, components of the piRNA pathway.

### Individual mouse cells support expression of multiple types of transposable elements

Our results show that individual MCF7 cells support expression of multiple L1 loci (Fig. 2C and 3C). Mouse genomes contain multiple currently active L1 subfamilies. Thus, we considered whether individual mouse cells support expression of multiple L1 subfamilies. Analysis of mouse L1 A, F, G_f_, and T_f_ subfamilies determined that all sperm cell types support expression of these subfamilies with Round Spermatids having the highest number of expressed L1 loci from the G_f_ and T_f_ subfamilies, the youngest and most active of the mouse L1 subfamilies (Additional file 7 A). Additionally, we identified that in Round Spermatids, 155 cells (46%) expressed at least two different L1 subfamilies (Additional file 7 B) and 7 (2.1%) Round Spermatids express at least one L1 locus from each active L1 subfamily (Additional file 7 B). These data show that similar to individual human breast cancer cells, cells in mouse testis also support expression of multiple L1 loci from the same or different subfamilies.

Mouse genomes contain different families of transposable elements that are active. To determine whether individual mouse testis cells express multiple families of mobile elements, we measured LTR expression in scRNA-Seq data of mouse testes. Bulk RNA-Seq analysis was performed on two 2 mo mouse datasets and expression from 143 LTRs was manually validated. LTR elements were included in our analysis if they were greater than 2 kb in length and received at least 10 aligned reads. Expression from 11 LTR elements (5 MMERVK, 3 IAP, and 3 MURVY) that were manually validated to be expressed in bulk RNA-Seq were analyzed in scRNA-Seq datasets (Additional file 17 and Additional file 7). Bulk RNA-Seq and scRNA-Seq of Mouse 1 and 2 testes shared the expression of 4 out of the 11 LTRs (Additional file 7 C). L1 and LTR element co-expression was detected in 1 Spermatogonia cell, 2 Spermatocytes, 14 Round Spermatids, and 2 Elongating Spermatids in Mouse 1 (Additional file 7 D1). L1 and LTR element co-expression was also detected in 17 Spermatocytes, 1 Round Spermatid, and 1 Elongating Spermatid in Mouse 2 (Additional file 7 D2). This analysis shows L1 and LTR elements are co-expressed in a subset of mouse Spermatocytes and Round Spermatids, the cell types with the highest L1 expression levels (Fig. 4 and Additional file 5).

### Mouse and human testes support similar L1 expression patterns

The increased L1 mRNA expression in mouse testes and round spermatids as well as the high levels of similarity in L1 loci expressed between testes taken from different mice led us to investigate whether similar patterns of L1 cell-type and locus specificity are conserved in human testes [10]. To determine whether there is a similar agreement as to which L1 loci are expressed in human testes from unrelated individuals, we performed bulk RNA-Seq using RNA extracted from testes samples obtained from two 20 yo donors followed by our previously reported L1 RNA-Seq analysis [12, 20, 30]. This approach identified 114 L1 loci that were expressed in testes samples collected from the two donors (Additional file 9 A). Of the 114 expressed L1 loci, 83% (95 of 114 L1 loci) were shared between the two unrelated 20 yo donors, demonstrating that human testes exhibit reproducible L1 expression patterns between biological replicates, similar to our previous study that showed testes collected from different mice shared 85% of expressed L1 loci (Additional file 5 A) [10]. L1 loci identified to be expressed in bulk RNA-Seq were then used to guide SCIFER analysis of scRNA-Seq datasets generated using testis samples from 24 yo and 25 yo donors, which share 77% (79 of 102) of expressed L1 loci (Additional file 9 B).

First, we confirmed proper cell-type clustering in the testis datasets from 24 yo and 25 yo donors by quantifying expression of testis cell-type-specific markers Prm1, Spag6, Tnp1, and TNP2. Human Prm1 expression, like mouse Prm1 expression, was detected at progressively increasing levels in Round, Elongating, and Condensing Spermatids (Additional file 17 and Additional file 8 A1 and C1). Spermatocytes, Round Spermatids, and Elongating Spermatids were observed to have high expression levels of Spag6 which contributes to sperm motility and maintenance of sperm structure in mature sperm (Additional file 17 and Additional file 8 A2 and C2) [58]. Elongating and Condensing Spermatids exhibited high expression levels of Tnp1 and Tnp2, genes encoding proteins involved in the exchange of histones for protamines during spermatid maturation (Additional file 17 and Additional file 8 A3, A4, C3, C4) [59].

**Fig. 5:**
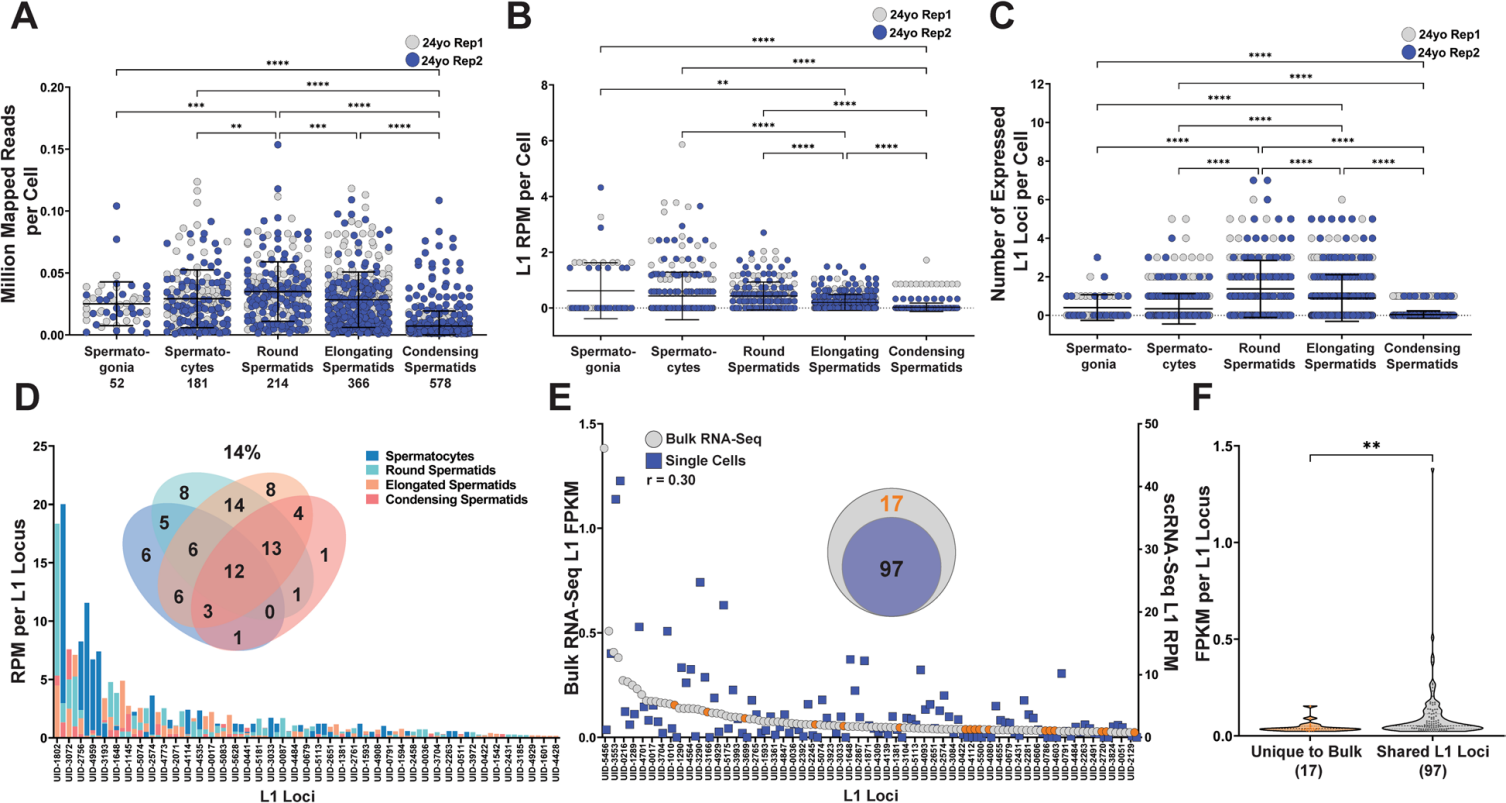
SCIFER detects cell type specific L1 mRNA expression in human testis. **A**. The number of million mapped reads per cell for each cell type is shown in the individual value plot for two technical replicates of the testis sample from a 24 yo donor. The technical replicates are indicated as gray and blue (ANOVA, **, < 0.005, ***, < 0.0005, ****, < 0.0001). **B**. L1 mRNA expression quantified by RPM for each cell is shown in the individual value plot for two technical replicates of the testis sample from a 24 yo donor. The technical replicates are indicated as gray and blue (ANOVA, **, < 0.005, ****, < 0.0001). **C**. The number of expressed L1 loci per cell in each cluster is shown in the individual value plot for two technical replicates of the testis sample from a 24 yo donors. The technical replicates are indicated as gray and blue (ANOVA, ****, < 0.0001). **D**. The L1 RPM values for L1 loci indicated on the X-axis are shown for each sperm cell type in the combined technical replicates of the testis sample from a 24 yo donor. The Venn Diagram shows the number of expressed L1 loci shared by the sperm cell types with the percentage of shared expressed L1 loci indicated above the diagram. **E**. The L1 FPKM values for bulk RNA-Seq (left y-axis) and L1 RPM values for scRNA-Seq (right y-axis) of human testis are shown in the dot-plot. Orange circles indicate L1 loci with detected expression in the bulk dataset that were not detected to be expressed in scRNA-Seq. The nested Venn diagram shows the number of shared expressed L1 loci from the bulk and scRNA-Seq datasets. **F**. The violin plot shows the FPKM values for expressed L1 loci unique to the bulk dataset and those shared between bulk and scRNA-Seq (Welch’s *t*-test, **, < 0.005)

Cell type-specific analysis of two technical replicates of scRNA-Seq from the testis of a 24 yo donor found that the number of mapped reads per cell was the highest in Round Spermatids (0.035) and Spermatocytes (0.029) compared to Spermatogonia (0.025), Elongated Spermatids (0.28), and Condensing Spermatids (0.007) (Fig. 5A). SCIFER analysis determined that on average Spermatogonia, Spermatocytes, and Round Spermatids supported the highest levels of L1 expression per cell (average L1 RPM per cell = 0.62, 0.43, and 0.43, respectively Fig. 5B) and Round Spermatids express, on average 1.2 L1 loci per cell, the highest number compared to the other cell types (Fig. 5C). Graphs that include Macrophages, Endothelial, Myoid, Sertoli, and Leydig cells from the 24 yo donor are presented in Additional file 9 C-E. Round Spermatids were confirmed to have significantly higher L1 expression levels compared to Condensing Spermatids using Seurat v4.0.5 (Wilcoxon rank sum, *P* = 3.68E-16, Additional file 8 D). To further confirm our results, we also SCIFERed scRNA-Seq from the testis of a 25 yo human donor. In this dataset, Round Spermatids had on average the highest number of reads per cell (0.022 million mapped reads per cell, Additional file 9 F), Spermatogonia had the highest levels of L1 expression per cell out of the sperm cell types (0.88 L1 RPM, Additional file 9 G), and Round Spermatids had the highest number of expressed L1 loci per cell (0.59 L1 loci expressed per cell, Additional file 9 H). These findings show that L1 expression patterns are somewhat conserved in mice and humans with Round Spermatids and Spermatogonia supporting high levels of L1 expression compared to the other cell types in Spermatogenesis. Similarly, while the number of L1 loci expressed is significantly greater in mouse Round Spermatids compared to human Round Spermatids, the average number of L1 loci per cell is 2.4 and 1.2, respectively (Welch’s *t*-test, *P* < 0.0001, Fig. 4C and 5C). Although this comparison is likely reflective of the biological differences and similarities between the two species, the accurate quantification of the trends detected in our studies can only be determined using datasets of the same sequencing depth. Based on our findings with MCF7 cells (Fig. 3), datasets from mouse and human testes with higher sequencing depth than the datasets analyzed here would be even more informative (Fig. 1B).

To determine the extent of heterogeneity of L1 expression among different sperm cell types, we compared the levels of individual L1 locus expression (Fig. 5D, graph) and the distribution of expressed L1 (Fig. 5D, Venn diagram) in different human testis sperm cell types. Different testis cell types in the 24 yo donor shared 14% (12 out of 88) of expressed L1 loci (Fig. 5D, Venn diagram). 27% (22 out of 81) of expressed L1 loci were shared between different cell types in the testis of the 25 yo donor (Additional file 9 I). By comparing the expressed L1 loci detected in bulk RNA-Seq of the testes from 20 yo donors to the expressed L1 loci detected in scRNA-Seq of the testis from a 24 yo donor, 85% (97 of 114) L1 loci expressed in bulk were expressed in the scRNA-Seq dataset (Fig. 5E). The 17 L1 loci unique to the bulk RNA-Seq dataset had a significantly lower FPKM compared to the L1 loci shared between the two datasets (*P =* 0.0032, Fig. 5F). We also performed a comparison between the bulk RNA-Seq from two 20 yo donors and scRNA-Seq from the 25 yo donor and found that 74% (84 of 114) of expressed L1 loci were shared between the bulk and single cell RNA-Seq datasets. The 30 L1 loci unique to bulk RNA-Seq had a significantly lower FPKM level than the L1 loci shared between bulk and single cell RNA-Seq (Welch’s t-test, *P* = 0.0035, Additional file 9 K). The number of shared L1 loci between different cell types in human testes is higher than in mouse testes (27–14% vs. 2–0%) potentially due to the higher number of Round Spermatids (499 cells) in mouse vs. Round Spermatids in human (214 cells). This is likely a reflection of the biological differences in the relative cell composition between mouse and human testes. Despite this difference, SCIFER detected expression from 85% of the L1 loci expressed in bulk RNA-Seq of samples from human testes in scRNA-Seq datasets.

### Expression patterns of genes restricting L1 expression and translation in human testis

To understand expression patterns of genes relevant to the L1 replication cycle, Seurat analysis [60] was used to determine the expression levels of genes previously identified to be involved in transcriptional and post-transcriptional regulation of L1 [50]. We examined expression of Dnmt1, Mecp2, Kdm1a, Trim28, and Ercc4, nuclear factors involved in the epigenetic regulation of L1 expression as well as Rnaseh2b, a gene involved in post-transcriptional regulation of L1 [50, 61]. In general, the same patterns of expression were observed for these genes in testes samples from the 24 yo and 25 yo donors. Spermatocytes expressed significantly higher levels of Dnmt1, Mecp2, and Kdm1a compared to Round, Elongating, and Condensing Spermatids in testes samples collected from 24 yo and 25 yo donors (Additional file 17, Additional file 10 A1–3 and B1–3). Spermatocytes and Round Spermatids express the highest levels of Trim28 in 24 yo and 25 yo testes compared to the other cell types (Additional file 17, Additional file 10 A5 and B5). Ercc4 expression was higher in Spermatocytes compared to Spermatids in both donors (*P <* 0.0001 (*P =* 0.017) (*P <* 0.0001) (Additional file 17, Additional file 10 A6 and B6). Rnaseh2b expression was significantly higher in Spermatocytes compared to Round, Elongating and Condensing Spermatids (Additional file 17, Additional file 10 A8 and B8). These gene expression patterns provide preliminary evidence that, in general, L1 inhibitory genes analyzed in this study peak in expression in Spermatocytes and their expression declines in Round, Elongating, and Condensing Spermatids. This peak in expression of genes that inhibit L1 expression corresponds to the peak in L1 expression observe in human Spermatocytes and Round Spermatids.

## Discussion

Using RNA-Seq to measure the expression of L1 elements derived from their own promoter is technically challenging, both because of difficulties properly aligning short-read sequences to a single, specific locus and the high levels of passive inclusion of L1 sequences in other transcripts that create high levels of background [10, 16–18, 20–22]. These difficulties are exacerbated by single-cell RNA-Seq procedures (scRNA-Seq) because the dominant method for measuring gene expression using 10X Chromium Single Cell 3′ Genomics Technology strongly targets sequencing to the 3′ end of mRNAs (Additional file 1). Targeting the 3′ end for sequencing limits discernment of authentic expression from passive expression by losing resolution of the 5′ end of the mRNA and transcriptional status of upstream regions [13, 20, 21, 30]. Furthermore, standard 10X Genomics scRNA-Seq analysis performs alignment with undefined stringency exclusively to the transcriptome whereas detection of L1-locus-specific expression requires alignment to the genome. To overcome these problems, we introduce SCIFER (**S**ingle **C**ell **I**mplementation to **F**ind **E**xpressed **R**etrotransposons), which allows unique mapping of scRNA-Seq reads to the genome while retaining cell-specific barcodes. SCIFER analysis also includes validating L1 expression detected in scRNA-Seq with the analysis of L1 expression in bulk RNA-Seq from a matching tissue sample using our existing approach for identifying expressed L1 mRNAs from individual sites, while eliminating those that are passively expressed [12, 20, 30]. This is a significant technical advancement given that the use of 10X software alone for TE analysis generates results that are biologically meaningless because it exponentially inflates the levels of L1 expression and the number of expressed L1 loci (Fig. 2F and G).

We validated SCIFER as a method for measuring L1 expression in single cells by analyzing a scRNA-Seq dataset generated from a pooled sample of MCF7 and HEK293 cells to determine whether our method was sensitive enough to detect the same loci as seen in the bulk RNA-Seq studies [29]. Our analysis showed that we were able to detect 93% of the L1 loci expressed in bulk RNA-Seq of MCF7 cells using the scRNA-Seq data (Fig. 2E). The L1 loci ‘missed’ by SCIFER were expressed at low levels in bulk RNA-Seq (Fig. 2E and Additional file 3D) and most likely fell below detection thresholds based on either poor mappability near the 3′ end or statistical fluctuation. A similar observation was made for ‘missed’ L1 loci when L1 expression was analyzed in mouse and human testes (Figs. 4F and 5F, Additional file 9 K). Thus, this approach can identify almost all the reasonably expressed L1 loci. It is clear, however, that detectability begins to fall off with lower reads/cell and that the threshold for detection will be higher in those cases (Fig. 3A and B and Additional file 3). SCIFER analysis of MCF7 cells with similar sequencing depth showed that cells differ in the number and identity of L1 loci expressed (Fig. 3C) with L1Hs loci expressed in a small subset of the cell population (Fig. 3B). While this cell-to cell variability in L1 expression between cells of similar sequencing depth could be due to sequencing drop-out, it also aligns with the report of differences in the number of L1 retrotransposition events between cells from adenocarcinoma tumors [48].

These findings have important implications for experimental design and comparative analysis of L1 expression in single cells. For example, comparisons between scRNA-Seq with significantly different average sequencing depth per cell are likely to produce artifacts regarding the number of expressed L1 loci per cell and the number of cells expressing specific L1 loci. However, both High and Low coverage datasets can be used for organ-derived studies to identify relative contribution of different cell types to L1 expression and the diversity and cell type specificity of expressed L1 loci in a specific organ (Fig. 2 and Additional file 3).

Our single-cell analysis also provides evidence that SCIFER can be adapted for detection of other repetitive sequences and assist in answering outstanding questions regarding mobile element biology. It has been proposed that L1 promoter evolution is driven in part by co-expression of different L1 subfamilies, which leads to their competition for cellular transcription factors and other transcriptional machinery [62–65]. Using SCIFER to analyze mouse testis scRNA-Seq, we observe that not only can the same cell express multiple L1 loci from the same, or different, subfamilies (Additional file 7 A and B), but that individual cells can also express both L1 elements and LTR elements (Additional file 7 C and D). While these results establish that some cells support expression from multiple types of mobile elements and multiple L1 subfamilies, they do not distinguish whether these levels of co-expression are sufficient to drive promoter competition or are a consequence of this competition with most cells expressing one L1 subfamily. We tested SCIFER on a complex mix of cells using mouse testes, which represent the organ with the highest detected level of L1 mRNA expression in mouse [10]. We found that Round Spermatids express the highest levels of L1 mRNA and the highest number of L1 loci compared with the other cell types and that this phenomenon was independent of differences in sequencing depth between cell types (Fig. 4A-D). Just like in the cell lines, we also found that SCIFER detected the vast majority of loci found in the bulk RNA-Seq analysis (Fig. 4 E and F). This evidence taken together with recent reports of epigenetic signatures driving L1 expression [29, 66] suggests epigenomic characteristics unique to Round Spermatids, such as the initiation of histone-protamine exchange (Additional file 4 A2 and B2), may facilitate an increase in L1 expression during this stage of Spermatogenesis.

We also performed SCIFER analysis on human testes to observe whether L1 expression patterns in testes are conserved between mice and humans. Bulk RNA-Seq of human testes from two unrelated 20 yo donors showed a high level of similarity in the L1 loci expressed (83%, Additional file 9 A), similar to the consistency in L1 loci shared between the same organ taken from different mice demonstrated in a previous study [10]. Although human Round Spermatids, like in mouse, express the highest number of L1 loci (Fig. 5C), Spermatogonia in the human datasets had higher average L1 RPM per cell than Round Spermatids (Fig. 5B). Despite observing comparable levels of L1 expression in multiple cell types in human testes, we still see cell-type specific expression of L1 loci with only 14–27% of expressed L1 loci shared between human Spermatocytes, Round Spermatids, Elongating Spermatids, and Condensing Spermatids (Fig. 5D and Additional file 9 I). This is consistent with the very high level of tissue specificity observed for L1 expression in mice [10]. It is also consistent with the cell-type specific epigenetic regulation seen for L1 mRNA expression [13, 29]. Furthermore, analysis of expression of genes involved in DNA methylation and the piRNA pathway showed that they peak in expression in Round Spermatids, the cell type that has the highest L1 expression in mouse testes, followed by downregulation (Additional file 6). In human testes, several genes involved in DNA methylation and RNA interference pathways peak in expression in spermatocytes and generally decrease in expression in the transition between Spermatocytes and Round Spermatids, the two cell types with the highest L1 expression (Additional file 10).

A technical limitation of SCIFER is that it only allows the detection of authentic L1 mRNA expression from loci that are detected first in the bulk RNA-Seq. Thus, if there is a cell type that is relatively rare in a tissue, expressed loci in those cells may be diluted so much in the bulk RNA-Seq that they are not detected. This limitation could be overcome by utilizing any procedure that carries out full-length RNA-Seq analysis from individual cells, rather than the strongly 3′-biased 10X Genomics procedure. For example, scRNA-Seq using long read sequencing, such as CELLO-Seq introduced by Berrens, et al., would increase the ability to unambiguously align L1 derived sequencing reads to young L1 loci [34]. However long-read sequencing is a costly method, and this approach does not allow analysis of L1 expression in scRNA-Seq data that is currently publicly available.

## Conclusions

Overall, SCIFER facilitates broad opportunities to understand the dynamics of L1 mRNA expression in real tissues and in response to various stimuli by significantly improving our ability to discover cell(s) of origin of L1 expression in different organs and species. Additionally, SCIFER has the ability to uncover evolutionary niches occupied by different classes of TEs or TE subfamilies, as well as to understand L1 impact on function and genome stability of single cells.

## Methods

### Cell culture

MCF7 (ATCC HTB-22) and HEK293-FRT-LacZeo cells were maintained in DMEM with high glucose (Gibco) supplemented with 10% fetal bovine serum (Gibco), sodium pyruvate, essential and nonessential amino acids, and L-glutamine. Cells at 100% confluency were detached using accutase (Innovative Cell Technologies) and resuspended in DPBS. MCF7 and HEK293-FRT-LacZeo cells were combined at a 9:1 ratio with 180 MCF7 and 20 HEK293 cells per uL. The single cell suspension was confirmed to be 84.4–83.1% viable prior to RNA preparation and sequencing.

### RNA sequencing of bulk human testes

Human testis bulk RNA samples from two 20-year-old (yo) donors were obtained from AMSBIO (product codes: CR562159 and CR562389). RNA was then poly-A selected prior to stranded, paired-end RNA sequencing using an Illumina NextSeq 2000 in the Tulane NextGen sequencing core.

### Single cell RNA sequencing

Single-cell RNA sequencing was carried out on individual cells using the 10X Genomics Chromium™ Single Cell 3′ Library & Gel Bead Kit v3 and 150 cycle kit by the Tulane NextGen sequencing core. Eight hundred fifty-three cells were sequenced and barcoded. Sequencing was carried out on an Illumina NextSeq 2000. Pooled MCF7 and HEK293-FRT-LacZeo cells were separated by aligning reads to an FRT-LacZeo genome. Cell barcodes with reads aligning to the FRT-LacZeo locus were classified as HEK293-FRT-LacZeo cells.

### Other scRNA-Seq datasets

The shallow sequenced MCF7 scRNA-Seq dataset and mouse and human testes scRNA-Seq datasets were obtained from NCBI SRA. The shallow sequenced MCF7 scRNA-Seq dataset is listed under SRR10018060. The mouse testes datasets are listed under SRR6129050 (Mouse 1) and SRR6129051 (Mouse 2). The human testes datasets are listed under SRR6860521 (24 yo) and SRR6860523 (25 yo).

### Bioinformatics analysis for bulk RNA-Seq

The strategy for detection of L1 mRNA used here has been described previously [30]. Briefly, we aligned bulk human testis RNA-Seq reads to the hg38 genome using Bowtie v0.12.8 and the following settings: -X600 to require concordant alignments, −m 1 to only align reads with one unique mapping position, −y to search exhaustively for each read’s best alignment, and -v 3 to allow 3 mismatches per alignment [43]. The resulting alignment file was then strand separated and the number of aligned reads corresponding to a list of full-length human L1 loci was counted using BEDTools v2.27.1. To find expression from mouse LTRs, RNA sequencing from two 2 mo mouse testes were aligned to the mm10 genome, as previously described, and alignments to 143 mouse LTR coordinates were quantified using BEDTools v2.27.1 coverage. Eleven LTRs were visually validated to be authentically expressed (Additional file 17) and were used to measure LTR expression in the scRNA-Seq dataset. Manual validation of L1 or LTR aligned reads in mouse and human samples was performed by visualizing alignments in IGV [67]. L1 or LTR loci were judged as true or false expression using previously established criteria [30] including inspection of upstream reads to determine whether the L1 locus expression originated from the L1 promoter.

### **S**ingle **C**ell **I**mplementation for **F**inding **E**xpressed **R**etrotransposons (SCIFER)

First, cells are submitted for 10X Chromium Single Cell 3′ RNA-Seq, sequencing reads containing cell-specific barcodes and read specific UMIs are generated, and reads are demultiplexed using the 10X Genomics cellranger mkfastq tool (Fig. 1A, step 1). Next, cell-specific barcodes and UMIs from the R1 (read 1) file are appended to the read header of the genomic read, R2 (read 2), file (Fig. 1A, step 2). Spaces are removed from the sequence header lines to retain barcodes during alignment. Reads are then aligned to the hg38 genome using Bowtie v0.12.8 and the settings described in *Bioinformatic Analysis for bulk RNA-Seq* (−m 1, −y, −v 3), resulting in unambiguous assignment of reads to their best genomic location (Fig. 1A, step 2). PCR duplicates are removed based on identical alignment coordinates using SAMtools rmdup. Alignments are then compared to a list of L1 coordinates. Barcodes and UMIs are extracted from reads that align to L1 sequences. Unique barcode-UMI pairs are kept along with one randomly selected alignment from duplicated barcode-UMI pairs. Cell-specific alignments are then extracted from the alignment file based on the list of de-duplicated barcode-UMIs detected for each alignment (Fig. 1A, step 2). Once alignments have been parsed for every cell, each individual cell alignment is strand separated and compared with a list of genome coordinates for L1 loci that have been visually validated as expressed in a matching bulk RNA-Seq dataset using BEDTools v2.27.1 coverage (Fig. 1A, step 2). Read alignments that occur in the same orientation as a validated L1 locus are counted as authentically expressed and the FPKM value for that locus is calculated (Fig. 1A, step 2). scRNA-Seq reads generated after the cellranger mkfastq step are also aligned using cellranger count to assign clusters. Downstream analysis with Seurat v4.0.5 quantifies differences in gene expression and identifies genes that are markers for different clusters and cell types (Fig. 1A, step 3) [60]. The command list to run SCIFER is in Additional file 11. The list of authentically expressed L1 loci from bulk RNA-Seq of MCF7 cells is in Additional file 17, the list for 2mo mouse testis is in Additional file 17, and the list for 20 yo human testis is in Additional file 17.

### Normalization of transcript reads

Bulk RNA-Seq sequencing reads were normalized by FPKM and scRNA-Seq sequencing reads were normalized by RPM. The FPKM was calculated by dividing the number of raw reads corresponding to a specific L1 locus by the number of million mapped reads in the sample multiplied by 6 (the length of L1), as previously reported [12, 30]. For RPM calculations using scRNA-Seq data, the number of million mapped reads was the sum of mapped reads for all cells in the corresponding cell cluster or cell type. The described formula for FPKM is shown below:

$$FPKM\ of\ L1\ locus\ z=\frac{\# of\ uniquely\ mapped\ reads\ to\ L1\ locus\ z\ in\ sample\ y}{million\ mapped\ reads\ in\ sample\ y\times 6}$$

The described formula for RPM is shown below:

$$RPM\ of\ L1\ locus\ z=\frac{\# of\ uniquely\ mapped\ reads\ to\ L1\ locus\ z\ in\ cluster\ y}{million\ mapped\ reads\ in\ cluster\ y}$$

### Bioinformatics analysis for scRNA-Seq

10X Chromium Single Cell 3′ Genomics data was demultiplexed and converted into fastq format using cellranger v3.1.0 mkfastq tool. The cellranger v3.1.0 count program was then used to analyze scRNA-Seq reads and alignment of reads to the appropriate reference genome to generate a gene expression matrix. The total number of L1 UMIs from SCIFER analysis were loaded into the gene expression matrix and analyzed using Seurat v4.0.5. The datasets were normalized (NormalizeData) and L1 expression was compared between cell clusters and cell types (FindAllMarkers).

### Gene expression analysis

Seurat v4.0.5 was used to compare gene expression levels between clusters and cell types [60]. Cells were considered for analysis if they contained < 5% of mitochondrial reads. Comparisons of gene expression between cell types (*n* = 2) were made using the normalized gene expression levels downloaded from the Seurat object and Welch’s *t*-tests.

### Statistical analysis

Bar graphs are presented with the mean and standard deviation bars. Data were analyzed by two-tailed Student’s *t*-test with Welch’s correction when making comparisons between two groups and by one-way ANOVA with multiple comparisons and “two-stage” Benjamini, Krieger, & Yekutiel for controlling the false discovery rate when making comparison within a *n* > 2 group. To measure linear correlation between two variables and determine the r value, a two-tailed Pearson correlation test was performed. Data presented from Seurat was analyzed using a non-parametric Wilcoxon rank-sum test with Bonferroni correction and the adjusted *P*-values from Bonferroni correction are plotted. Statistical analysis was performed with GraphPad Prism and Seurat v4.0.5.

## Supplementary Information

**Additional file 1. **Single cell RNA-Seq reads align to the 3′ end of genes and L1 loci. **A**. Alignment of scRNA-Seq reads (top), bulk RNA-Seq reads (middle), and DNA-Seq reads (bottom) to the L1 consensus sequence using Bowtie v0.12.8. The sequencing read scale is indicated in the bottom left corners. **B**. Alignment of MCF7 bulk RNA-Seq and MCF7 scRNA-Seq reads to the actin (ACTB) gene locus (top) and an L1 locus (bottom). Images were taken from IGV and the visible tracks include, from top to bottom, chromosome location indicated by the red line, scale in base pairs, mappability from a DNA-Seq samples aligned with the same bowtie settings used for RNA-Seq (see Methods), hg38 genes, L1 annotation (bottom only), Bulk RNA-Seq alignment, and scRNA-Seq alignment.

**Additional file 2. **Analysis of clustering and L1 expression in scRNA-Seq datasets **A**. A t-SNE plot of the combined High coverage MCF7 and HEK293 scRNA-Seq dataset. MCF7 or HEK293 cell clusters are indicated in the figure legend. **B**. Violin plots of the number of expressed genes per cell (left), RNA molecules per cell (middle), and percent of mitochondrial reads per cell (right) for each MCF7 cell cluster. **C**. The number of expressed L1 loci per cell for all High coverage MCF7 cell clusters is shown in the individual value plot (ANOVA, *, < 0.05, **, < 0.005, ****, < 0.0001). **D**. The L1 mRNA expression level per cell for all MCF7 clusters is shown in the individual value plot (ANOVA, ****, < 0.0001). **E**. The normalized expression levels for MCF7 Clusters 1, 3, and 4 from Seurat analysis are shown (Wilcoxon rank sum, **, *P* = 0.00046). **F**. The number of reads per HKG averaged between 3 MCF7 cells downsampled in 10% intervals is shown. **G**. The average number of expressed HKGs detected in 3 MCF7 cells is shown with decreasing read depth (100–10% of total cell reads). The black bars have significantly higher numbers of detected HKGs compared to the gray bars (Welch’s *t*-test, 100% vs. 40%, *P =* 0.013). The lightest gray bar (10%) indicates detection of less than half of expressed HKGs detected with 100% of reads. **H**. A correlation matrix of HKGs expressed in 10 MCF7 cells. The HKGs are listed at the top of the matrix and the 10 cells are listed along the left side. Filled boxes indicate that the HKG was expressed in the corresponding cell.

**Additional file 3. **Low depth sequencing reduces sensitivity of L1 mRNA expression detection in MCF7 single cells. **A**. A t-SNE plot of the Low coverage MCF7 scRNA-Seq dataset. Cell clusters are indicated in the figure legend. **B**. The number of million mapped reads per cell in each cluster is shown in the individual value plot. **C**. L1 mRNA expression quantified by RPM for each cell is shown in the individual value plot. **D**. The number of expressed L1 loci per cell in each cluster is shown in the individual value plot **E**. The L1 FPKM values for bulk RNA-Seq (left y-axis) and L1 RPM values for Low coverage MCF7 scRNA-Seq (right y-axis) are shown in the dot-plot. Orange circles indicate L1 loci with detected expression in the bulk dataset that were not detected to be expressed in scRNA-Seq. The nested Venn diagram shows the number of shared expressed L1 loci from the bulk and scRNA-Seq datasets. The violin plot shows the FPKM values for expressed L1 loci unique to the bulk dataset and those shared between bulk and scRNA-Seq (t-test, ****,< 0.0001).

**Additional file 4. **Expression patterns of genes involved in spermatogenesis in mouse testes. Cell types are abbreviated as follows: Spermatocytes (Spcyt), Round Spermatids (RS), Elongating Spermatids (ES), Condensing Spermatids (CS), Sertoli (Ser), Leydig (Ley). **A**. Mouse 1 gene expression patterns for TATA-Box Binding Protein Like-1 (TBPL1) (A1), Protamine (PRM1) (A2), and Transition Protein 1 (TNP1) (A3). **B**. Mouse 2 gene expression patterns for TBPL1 (B1), PRM1 (B2), and TNP1 (B3). **C**. The heat map shows the normalized expression patterns for each cell type and gene in Mouse 1 (top) and Mouse 2 (bottom). The asterisks indicate a significant change in gene expression in the cell type compared to the preceding cell type in the row (*P* < 0.0001). **D**. The normalized expression levels for Mouse 1 testis cell types from Seurat analysis are shown (Wilcoxon rank sum, *, *P* < 0.05, ****, *P* < 0.0001).

**Additional file 5. **SCIFER analysis of L1 mRNA expression in mouse testis biological replicates. **A**. The number of million mapped reads per cell for each testis cell type is shown for Mouse 1 (ANOVA,, *, < 0.05, **, < 0.005,, ***, < 0.0005, ****,< 0.0001). **B**. The number of million mapped reads per cell for each testis cell type is shown for Mouse 2 ANOVA,, ***, < 0.0005, ****,< 0.0001). **C**. L1 mRNA expression measured by RPM per cell for all testis cell types is shown in the scatter plot for Mouse 1 (ANOVA,****,< 0.0001). **D**. L1 mRNA expression measured by RPM per cell for all testis cells types is shown in the scatter plot for Mouse 2 (ANOVA, ***, < 0.0005, ****,< 0.0001). **E**. The number of expressed L1 loci per cell for each testis cell type is shown in the scatter plot for Mouse 1 (ANOVA, ***, < 0.0005, ****,< 0.0001). **F**. The number of expressed L1 loci per cell for each testis cell type is shown in the scatter plot for Mouse 2 (ANOVA, **, < 0.005, ****,< 0.0001). **G**. The L1 RPM values for L1 loci indicated on the X-axis are shown for each sperm cell type in Mouse 2. The Venn Diagram shows the number of expressed L1 loci shared by the sperm cell types with the percentage of shared expressed L1 loci indicated above the diagram. **H**. L1 mRNA expression compared between Mouse 1 and Mouse 2 for Spermatocytes (SC) (H1), Round Spermatids (RS) (H2), Elongating Spermatids (ES) (H3), and Condensing Spermatids (CS) (H4).

**Additional file 6. **Expression patterns of genes involved in limiting L1 expression and integration in mice. Cell types are abbreviated as follows: Spermatocytes (Spcyt), Round Spermatids (RS), Elongating Spermatids (ES), Condensing Spermatids (CS), Sertoli (Ser), Leydig (Ley). **A**. Mouse 1 gene expression patterns for Phospholipase D Family Member 6 (PLD6) (A1), Heat Shock Protein 90 Alpha Family Class A Member 1 (HSP90AA1) (A2), and Ubiquitin Like with PHD And Ring Finger Domains 1 (UHRF1) (A3). **B**. Mouse 2 gene expression patterns for PLD6 (B1), HSP90AA1 (B2), and UHRF1 (B3). **C**. The heat map shows the normalized expression patterns for each cell type and gene in Mouse 1 (top) and Mouse 2 (bottom). The asterisks indicate a significant change in gene expression in the cell type compared to the preceding cell type in the row (*P* < 0.0001).

**Additional file 7. **Expression of LTRs and L1 subfamilies is non uniform across single cells in mouse testis. **A**. The number of expressed L1 loci per L1 subfamily is shown for Mouse 1. The L1 subfamilies are indicated in the legend. **B**. The number of Round Spermatids expressing L1 loci from each mouse L1 subfamily is shown in the Venn diagram. **C**. The number of reads per LTR locus is shown for bulk RNA-Seq, Mouse 1 Single Cells, and Mouse 2 Single Cells. The Venn diagram shows the number of expressed LTR loci shared between the three datasets. **D**. Cells expressing at least one LTR loci are shown for each cell type in Mouse 1 (D1) and Mouse 2 (D2). LTR expression is indicated with purple and L1 co-expression is indicated with teal.

**Additional file 8 **Expression patterns of genes involved in spermatogenesis in human testes. Cell types are abbreviated as follows: Spermatogonial Stem Cells (SSCs), Differentiating Spermatogonia (Diff Spg), Spermatocytes (Spcyt), Round Spermatids (RS), Elongating Spermatids (ES), Condensing Spermatids (CS), Macrophages (Macro), Endothelial (Endo), Myoid (Myo), and Sertoli (Ser). **A**. 24 yo Human Testis gene expression patterns for PRM1 (A1), SPAG6 (A2), TNP1 (A3), and TNP2 (A4). **B**. 25 yo Human Testis gene expression patterns for PRM1 (B1), SPAG6 (B2), TNP1 (B3), and TNP2 (B4). **C**. The heat map shows the normalized expression patterns for each cell type and gene in 24 yo Testis (top) and 25 yo Testis (bottom). The asterisks indicate a significant change in gene expression in the cell type compared to the preceding cell type in the row (*P* < 0.0001). **D**. The normalized expression levels for 24 yo testis cell types from Seurat analysis are shown (Wilcoxon rank sum, *P* < 0.0001).

**Additional file 9. **Analysis of L1 mRNA expression in human testes with bulk RNA-Seq and scRNA-Seq. **A**. The L1 FPKM values for L1 loci listed on the X-axis are shown for two 20 yo human testis bulk RNA-Seq samples. The number of expressed L1 loci shared between the two replicates is shown in the Venn diagram with the percentage of shared expressed loci indicated above the diagram. **B**. The L1 RPM values for L1 loci listed on the X-axis are shown for a 24 yo and a 25 yo human testis scRNA-Seq sample. The number of expressed L1 loci shared between the two replicates is shown in the Venn diagram with the percentage of shared expressed loci indicated above the diagram. **C**. The million mapped reads per cell for all cell types in the 24 yo scRNA-Seq dataset is shown in the scatter plot. Statistics are shown for sperm cells and sperm progenitor cell types only. **D**. The L1 mRNA expression level per cell for all 24 yo testis cell types is shown in the scatter plot. **E**. The number of expressed L1 loci per cell for all 24 yo testis cell types is shown in the scatter plot. **F**. The million mapped reads per cell for all cell types in the 24 yo scRNA-Seq dataset is shown in the scatter plot. Statistics are shown for sperm cells and sperm progenitor cell types only**. G**. The L1 mRNA expression level per cell for all 25 yo testis cell types is shown in the scatter plot. **H**. The number of expressed L1 loci per cell for all 25 yo testis cell types is shown in the scatter plot. **I**. The L1 RPM values for L1 loci indicated on the X-axis are shown for each sperm cell type in the 25 yo human testis sample. The Venn Diagram shows the number of expressed L1 loci shared by the sperm cell types with the percentage of shared expressed L1 loci indicated above the diagram. **J**. The L1 FPKM values for bulk RNA-Seq (left y-axis) and L1 RPM values for scRNA-Seq (right y-axis) of human testis are shown in the dot-plot. Orange circles indicate L1 loci with detected expression in the bulk dataset that were not detected to be expressed in scRNA-Seq. The nested Venn diagram shows the number of shared expressed L1 loci from the bulk and scRNA-Seq datasets. **K**. The violin plot shows the FPKM values for expressed L1 loci unique to the bulk dataset and those shared between bulk and scRNA-Seq (t-test, **,< 0.0021).

**Additional file 10. **Expression patterns of genes involved in limiting L1 expression and integration in humans. Cell types are abbreviated as follows: Spermatogonial Stem Cells (SSCs), Differentiating Spermatogonia (Diff Spg), Spermatocytes (Spcyt), Round Spermatids (RS), Elongating Spermatids (ES), Condensing Spermatids (CS), Macrophages (Macro), Endothelial (Endo), Myoid (Myo), and Sertoli (Ser). **A**. 24 yo Human Testis gene expression patterns for DNMT1 (A1), MECP2 (A2), KDM1A (A3), TP53 (A4), TRIM28 (A5), ERCC4 (A6), MOV10 (A7), and RNASEH2B (A8). **B**. 25 yo Human Testis gene expression patterns for DNMT1 (B1), MECP2 (B2), KDM1A (B3), TP53 (B4), TRIM28 (B5), ERCC4 (B6), MOV10 (B7), and RNASEH2B (B8). **C**. The heat map shows the normalized expression patterns for each cell type and gene in 24 yo Testis (top) and 25 yo Testis (bottom). The asterisks indicate a significant change in gene expression in the cell type compared to the preceding cell type in the row (*P* < 0.05).

**Additional file 11. **Directions for running **S**ingle **C**ell **I**mplementation to **F**ind **E**xpressed **R**etrotransposons (SCIFER).

**Additional file 12.** Coordinates of L1 loci identified to be expressed in bulk RNA-Seq of MCF7 cells and included in SCIFER analysis.

**Additional file 13.** Coordinates of LTR loci identified to be expressed in bulk RNA-Seq of 2 mo mouse testes and included in SCIFER analysis.

**Additional file 14.** Coordinates of L1 loci identified to be expressed in bulk RNA-Seq of 2 mo mouse testes and included in SCIFER analysis.

**Additional file 15.** Coordinates and FPKM of L1 loci identified to be expressed in bulk RNA-Seq of 20 yo human testes and included in SCIFER analysis.

**Additional file 16.** Results of comparing gene expression values in mouse testes using unpaired t-test with Welch’s correction.

**Additional file 17.** Results of comparing gene expression values in human testes using unpaired t-test with Welch’s correction.

## Data Availability

The raw sequencing files for human testis bulk RNA-Seq in this manuscript are available through BioProject ID: PRJNA809333. The raw sequencing files for MCF7 scRNA-Seq are available through GEO:XX.
