# Supplementary material for: SCIFER: approach for analysis of LINE-1 mRNA expression in single cells at a single locus resolution
Source: Mob DNA. 2022 Aug 26;13:21. doi: 10.1186/s13100-022-00276-0 (PMC9413895; doi:10.1186/s13100-022-00276-0)

Additional File 1

A

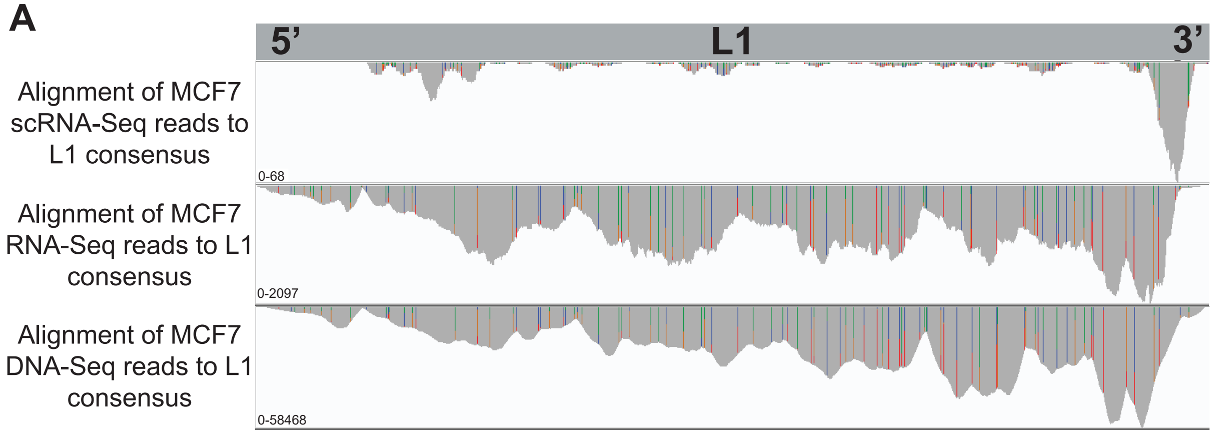

B

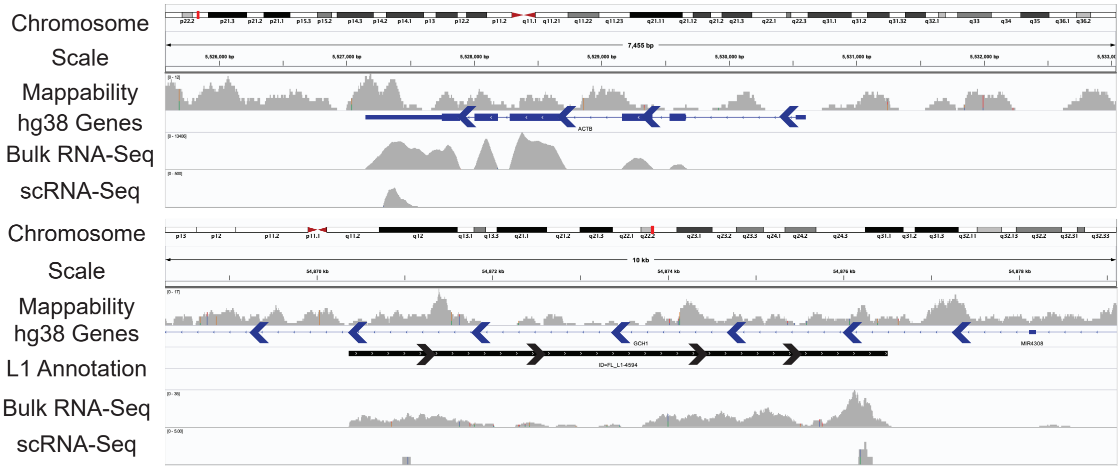

Supplement: Supplementary file 1 — Additional file 1. Single cell RNA-Seq reads align to the 3′ end of genes and L1 loci. A. Alignment of scRNA-Seq reads (top), bulk RNA-Seq reads (middle), and DNA-Seq reads (bottom) to the L1 consensus sequence using Bowtie v0.12.8. The sequencing read scale is indicated in the bottom left corners. B. Alignment of MCF7 bulk RNA-Seq and MCF7 scRNA-Seq reads to the actin (ACTB) gene locus (top) and an L1 locus (bottom). Images were taken from IGV and the visible tracks include, from top to bottom, chromosome location indicated by the red line, scale in base pairs, mappability from a DNA-Seq samples aligned with the same bowtie settings used for RNA-Seq (see Methods), hg38 genes, L1 annotation (bottom only), Bulk RNA-Seq alignment, and scRNA-Seq alignment. [file 13100_2022_276_MOESM1_ESM.pdf]
