# Supplementary material for: SCIFER: approach for analysis of LINE-1 mRNA expression in single cells at a single locus resolution
Source: Mob DNA. 2022 Aug 26;13:21. doi: 10.1186/s13100-022-00276-0 (PMC9413895; doi:10.1186/s13100-022-00276-0)

Additional File 2

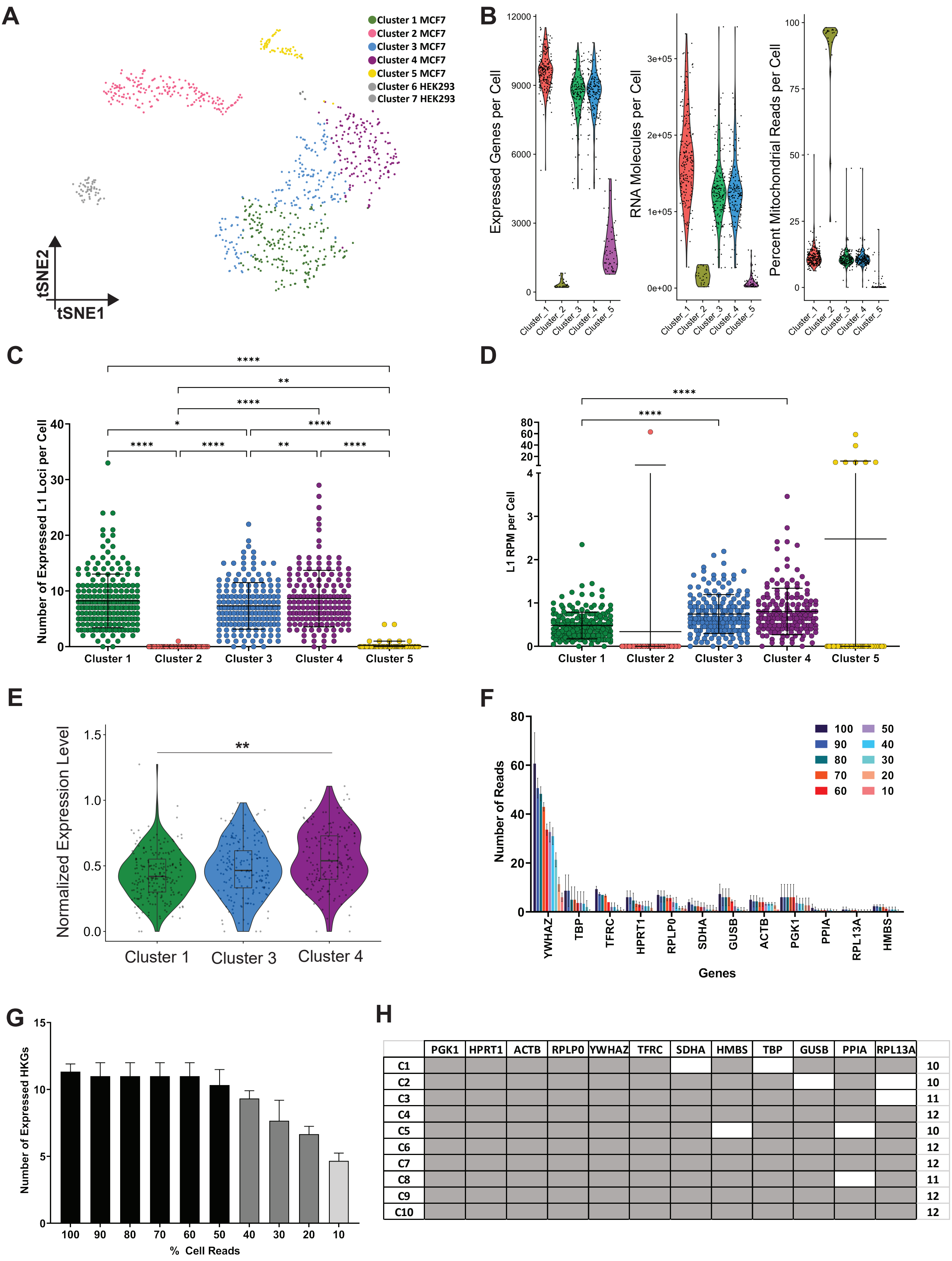

Supplement: Supplementary file 2 — Additional file 2. Analysis of clustering and L1 expression in scRNA-Seq datasets A. A t-SNE plot of the combined High coverage MCF7 and HEK293 scRNA-Seq dataset. MCF7 or HEK293 cell clusters are indicated in the figure legend. B. Violin plots of the number of expressed genes per cell (left), RNA molecules per cell (middle), and percent of mitochondrial reads per cell (right) for each MCF7 cell cluster. C. The number of expressed L1 loci per cell for all High coverage MCF7 cell clusters is shown in the individual value plot (ANOVA, *, < 0.05, **, < 0.005, ****, < 0.0001). D. The L1 mRNA expression level per cell for all MCF7 clusters is shown in the individual value plot (ANOVA, ****, < 0.0001). E. The normalized expression levels for MCF7 Clusters 1, 3, and 4 from Seurat analysis are shown (Wilcoxon rank sum, **, P = 0.00046). F. The number of reads per HKG averaged between 3 MCF7 cells downsampled in 10% intervals is shown. G. The average number of expressed HKGs detected in 3 MCF7 cells is shown with decreasing read depth (100–10% of total cell reads). The black bars have significantly higher numbers of detected HKGs compared to the gray bars (Welch’s t-test, 100% vs. 40%, P = 0.013). The lightest gray bar (10%) indicates detection of less than half of expressed HKGs detected with 100% of reads. H. A correlation matrix of HKGs expressed in 10 MCF7 cells. The HKGs are listed at the top of the matrix and the 10 cells are listed along the left side. Filled boxes indicate that the HKG was expressed in the corresponding cell. [file 13100_2022_276_MOESM2_ESM.pdf]
