# Supplementary figures and images for: SCIFER: approach for analysis of LINE-1 mRNA expression in single cells at a single locus resolution
Source: Mob DNA. 2022 Aug 26;13:21. doi: 10.1186/s13100-022-00276-0 (PMC9413895; doi:10.1186/s13100-022-00276-0)

Additional File 3

A

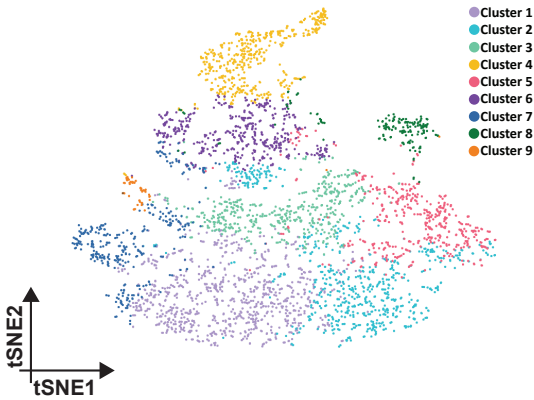

B

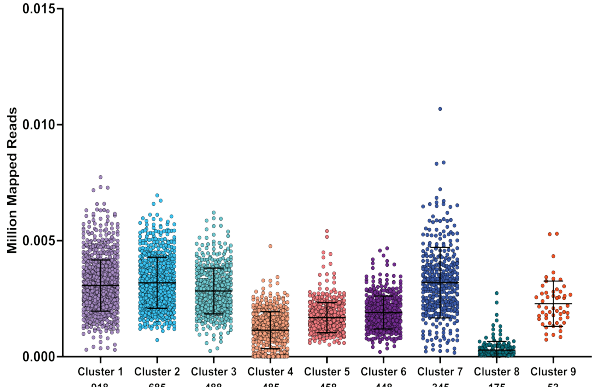

C

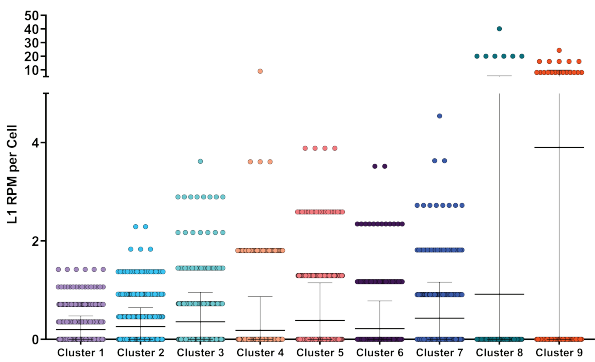

D

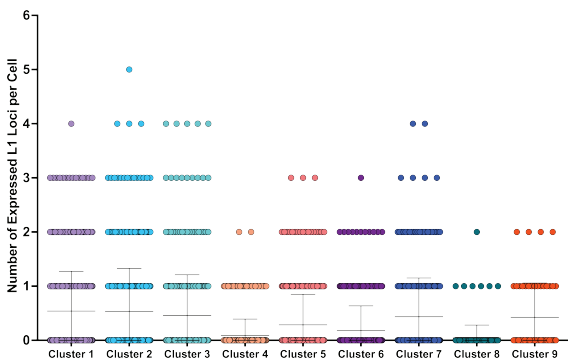

E

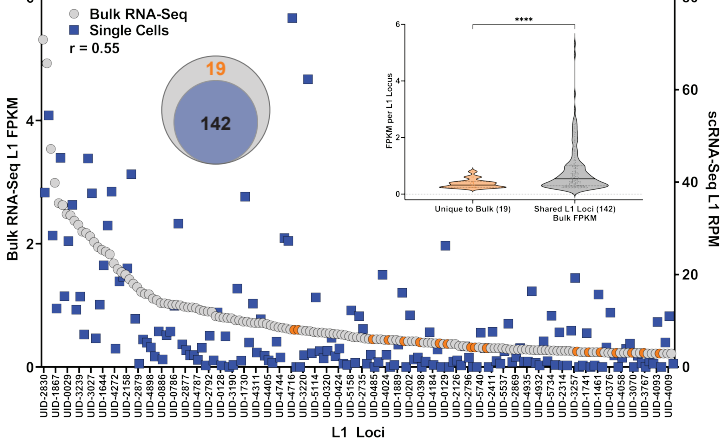

Supplement: Supplementary file 3 — Additional file 3. Low depth sequencing reduces sensitivity of L1 mRNA expression detection in MCF7 single cells. A. A t-SNE plot of the Low coverage MCF7 scRNA-Seq dataset. Cell clusters are indicated in the figure legend. B. The number of million mapped reads per cell in each cluster is shown in the individual value plot. C. L1 mRNA expression quantified by RPM for each cell is shown in the individual value plot. D. The number of expressed L1 loci per cell in each cluster is shown in the individual value plot E. The L1 FPKM values for bulk RNA-Seq (left y-axis) and L1 RPM values for Low coverage MCF7 scRNA-Seq (right y-axis) are shown in the dot-plot. Orange circles indicate L1 loci with detected expression in the bulk dataset that were not detected to be expressed in scRNA-Seq. The nested Venn diagram shows the number of shared expressed L1 loci from the bulk and scRNA-Seq datasets. The violin plot shows the FPKM values for expressed L1 loci unique to the bulk dataset and those shared between bulk and scRNA-Seq (t-test, ****,< 0.0001). [file 13100_2022_276_MOESM3_ESM.pdf]

Additional File 4

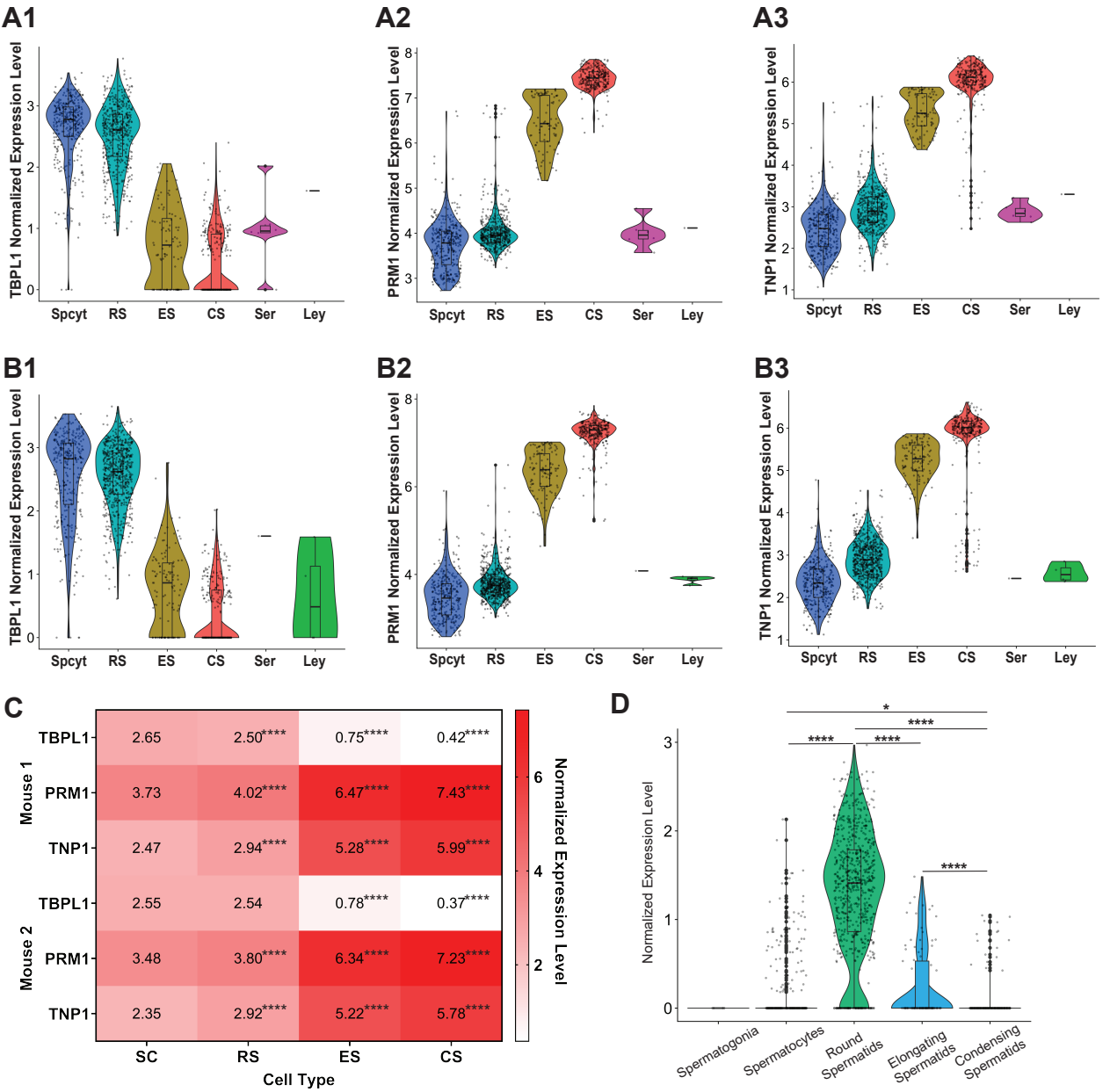

Supplement: Supplementary file 4 — Additional file 4. Expression patterns of genes involved in spermatogenesis in mouse testes. Cell types are abbreviated as follows: Spermatocytes (Spcyt), Round Spermatids (RS), Elongating Spermatids (ES), Condensing Spermatids (CS), Sertoli (Ser), Leydig (Ley). A. Mouse 1 gene expression patterns for TATA-Box Binding Protein Like-1 (TBPL1) (A1), Protamine (PRM1) (A2), and Transition Protein 1 (TNP1) (A3). B. Mouse 2 gene expression patterns for TBPL1 (B1), PRM1 (B2), and TNP1 (B3). C. The heat map shows the normalized expression patterns for each cell type and gene in Mouse 1 (top) and Mouse 2 (bottom). The asterisks indicate a significant change in gene expression in the cell type compared to the preceding cell type in the row (P < 0.0001). D. The normalized expression levels for Mouse 1 testis cell types from Seurat analysis are shown (Wilcoxon rank sum, *, P < 0.05, ****, P < 0.0001). [file 13100_2022_276_MOESM4_ESM.pdf]

Additional File 5

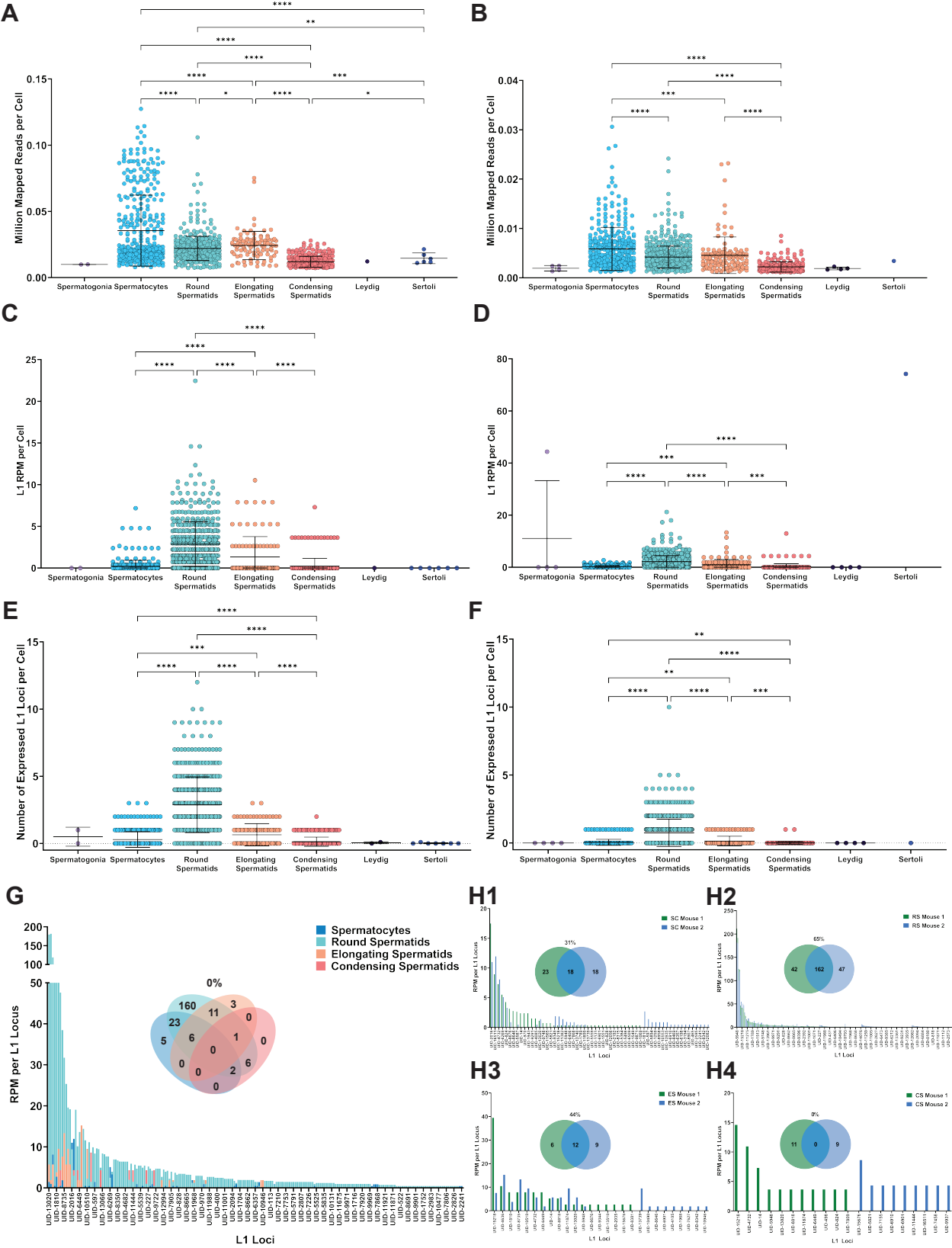

Supplement: Supplementary file 5 — Additional file 5. SCIFER analysis of L1 mRNA expression in mouse testis biological replicates. A. The number of million mapped reads per cell for each testis cell type is shown for Mouse 1 (ANOVA,, *, < 0.05, **, < 0.005,, ***, < 0.0005, ****,< 0.0001). B. The number of million mapped reads per cell for each testis cell type is shown for Mouse 2 ANOVA,, ***, < 0.0005, ****,< 0.0001). C. L1 mRNA expression measured by RPM per cell for all testis cell types is shown in the scatter plot for Mouse 1 (ANOVA,****,< 0.0001). D. L1 mRNA expression measured by RPM per cell for all testis cells types is shown in the scatter plot for Mouse 2 (ANOVA, ***, < 0.0005, ****,< 0.0001). E. The number of expressed L1 loci per cell for each testis cell type is shown in the scatter plot for Mouse 1 (ANOVA, ***, < 0.0005, ****,< 0.0001). F. The number of expressed L1 loci per cell for each testis cell type is shown in the scatter plot for Mouse 2 (ANOVA, **, < 0.005, ****,< 0.0001). G. The L1 RPM values for L1 loci indicated on the X-axis are shown for each sperm cell type in Mouse 2. The Venn Diagram shows the number of expressed L1 loci shared by the sperm cell types with the percentage of shared expressed L1 loci indicated above the diagram. H. L1 mRNA expression compared between Mouse 1 and Mouse 2 for Spermatocytes (SC) (H1), Round Spermatids (RS) (H2), Elongating Spermatids (ES) (H3), and Condensing Spermatids (CS) (H4). [file 13100_2022_276_MOESM5_ESM.pdf]

Additional File 6

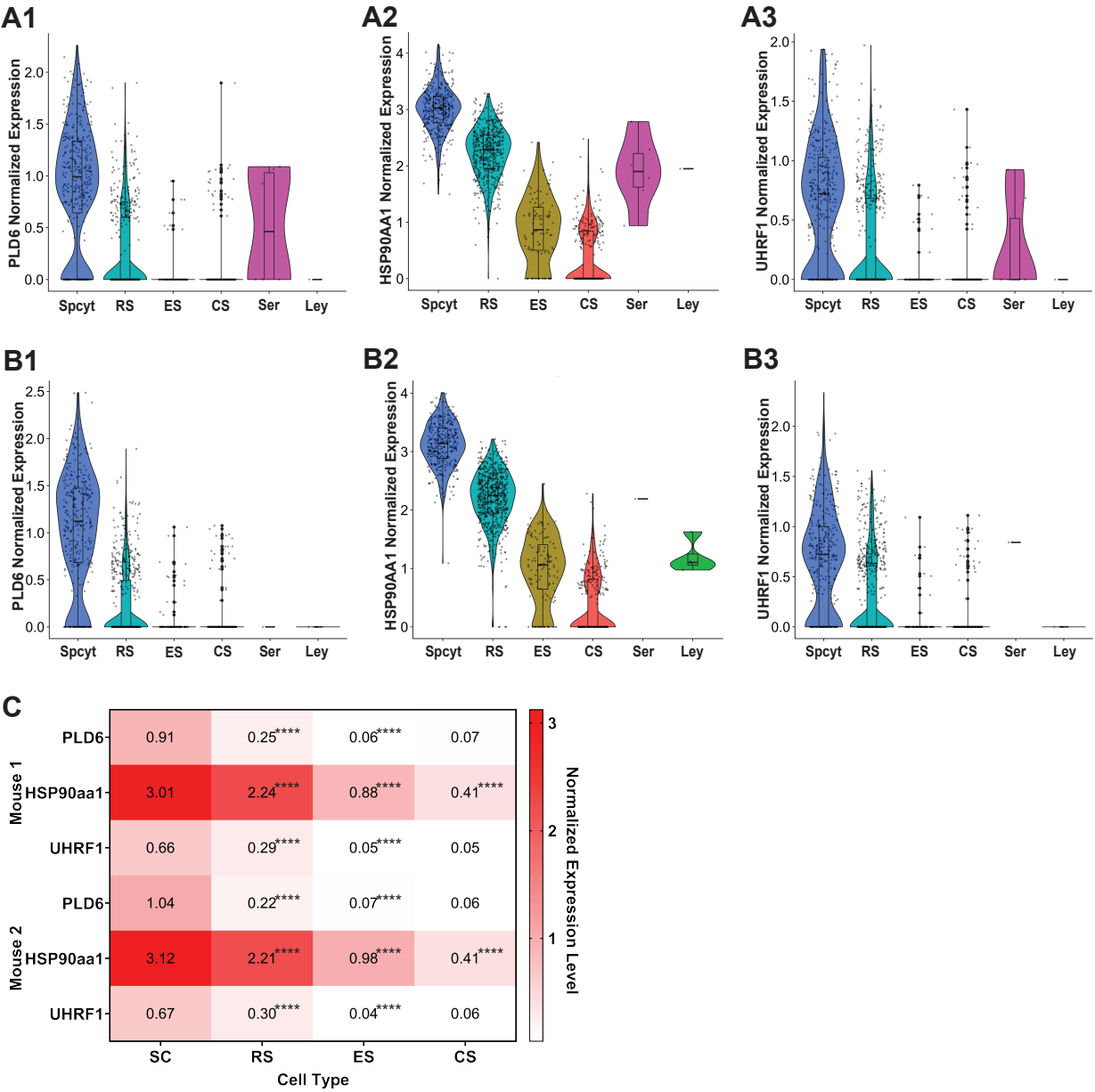

Supplement: Supplementary file 6 — Additional file 6. Expression patterns of genes involved in limiting L1 expression and integration in mice. Cell types are abbreviated as follows: Spermatocytes (Spcyt), Round Spermatids (RS), Elongating Spermatids (ES), Condensing Spermatids (CS), Sertoli (Ser), Leydig (Ley). A. Mouse 1 gene expression patterns for Phospholipase D Family Member 6 (PLD6) (A1), Heat Shock Protein 90 Alpha Family Class A Member 1 (HSP90AA1) (A2), and Ubiquitin Like with PHD And Ring Finger Domains 1 (UHRF1) (A3). B. Mouse 2 gene expression patterns for PLD6 (B1), HSP90AA1 (B2), and UHRF1 (B3). C. The heat map shows the normalized expression patterns for each cell type and gene in Mouse 1 (top) and Mouse 2 (bottom). The asterisks indicate a significant change in gene expression in the cell type compared to the preceding cell type in the row (P < 0.0001). [file 13100_2022_276_MOESM6_ESM.pdf]

Additional File 7

A

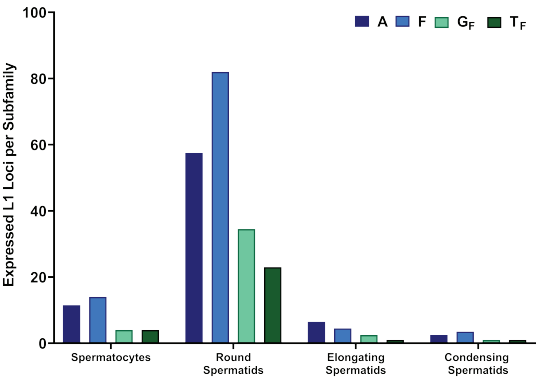

B

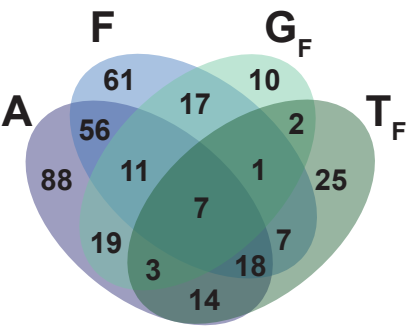

C

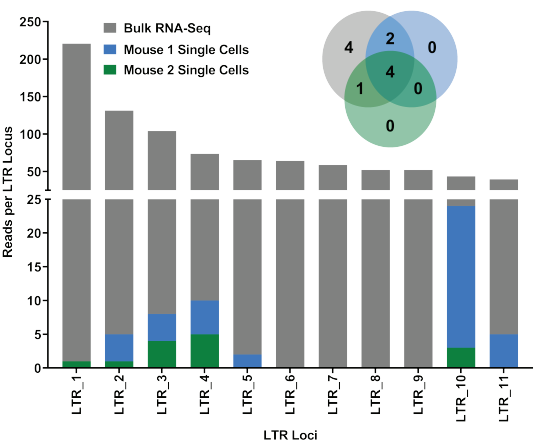

D1

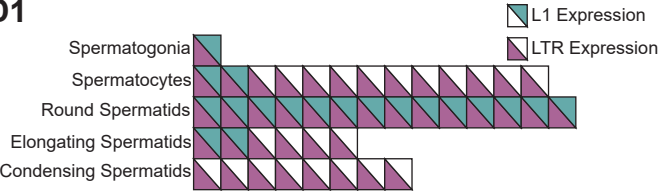

D2

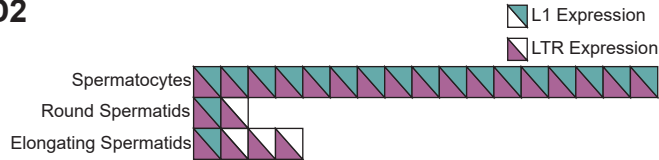

Supplement: Supplementary file 7 — Additional file 7. Expression of LTRs and L1 subfamilies is non uniform across single cells in mouse testis. A. The number of expressed L1 loci per L1 subfamily is shown for Mouse 1. The L1 subfamilies are indicated in the legend. B. The number of Round Spermatids expressing L1 loci from each mouse L1 subfamily is shown in the Venn diagram. C. The number of reads per LTR locus is shown for bulk RNA-Seq, Mouse 1 Single Cells, and Mouse 2 Single Cells. The Venn diagram shows the number of expressed LTR loci shared between the three datasets. D. Cells expressing at least one LTR loci are shown for each cell type in Mouse 1 (D1) and Mouse 2 (D2). LTR expression is indicated with purple and L1 co-expression is indicated with teal. [file 13100_2022_276_MOESM7_ESM.pdf]

Additional File 8

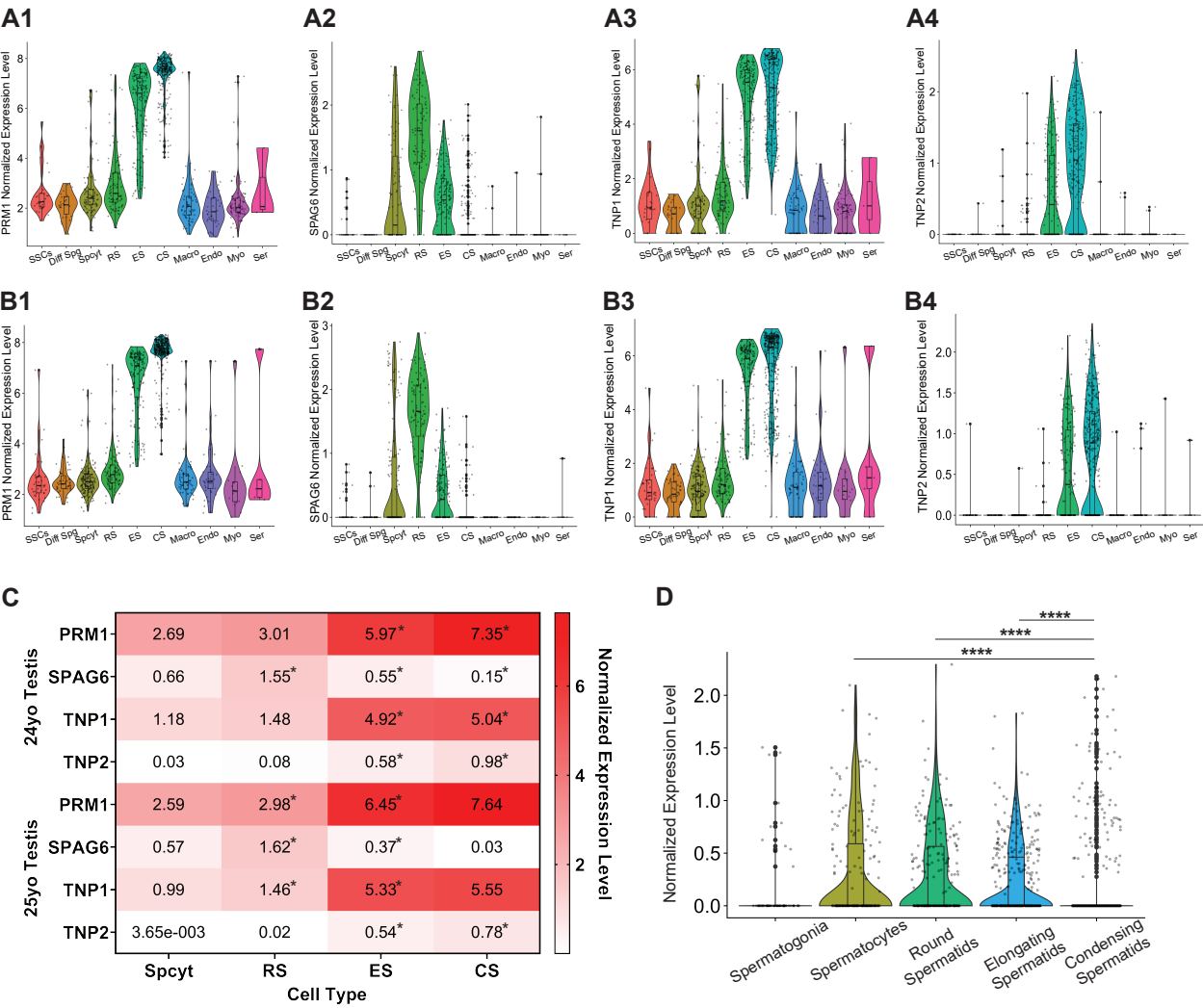

Supplement: Supplementary file 8 — Additional file 8 Expression patterns of genes involved in spermatogenesis in human testes. Cell types are abbreviated as follows: Spermatogonial Stem Cells (SSCs), Differentiating Spermatogonia (Diff Spg), Spermatocytes (Spcyt), Round Spermatids (RS), Elongating Spermatids (ES), Condensing Spermatids (CS), Macrophages (Macro), Endothelial (Endo), Myoid (Myo), and Sertoli (Ser). A. 24 yo Human Testis gene expression patterns for PRM1 (A1), SPAG6 (A2), TNP1 (A3), and TNP2 (A4). B. 25 yo Human Testis gene expression patterns for PRM1 (B1), SPAG6 (B2), TNP1 (B3), and TNP2 (B4). C. The heat map shows the normalized expression patterns for each cell type and gene in 24 yo Testis (top) and 25 yo Testis (bottom). The asterisks indicate a significant change in gene expression in the cell type compared to the preceding cell type in the row (P < 0.0001). D. The normalized expression levels for 24 yo testis cell types from Seurat analysis are shown (Wilcoxon rank sum, P < 0.0001). [file 13100_2022_276_MOESM8_ESM.pdf]

Additional File 9

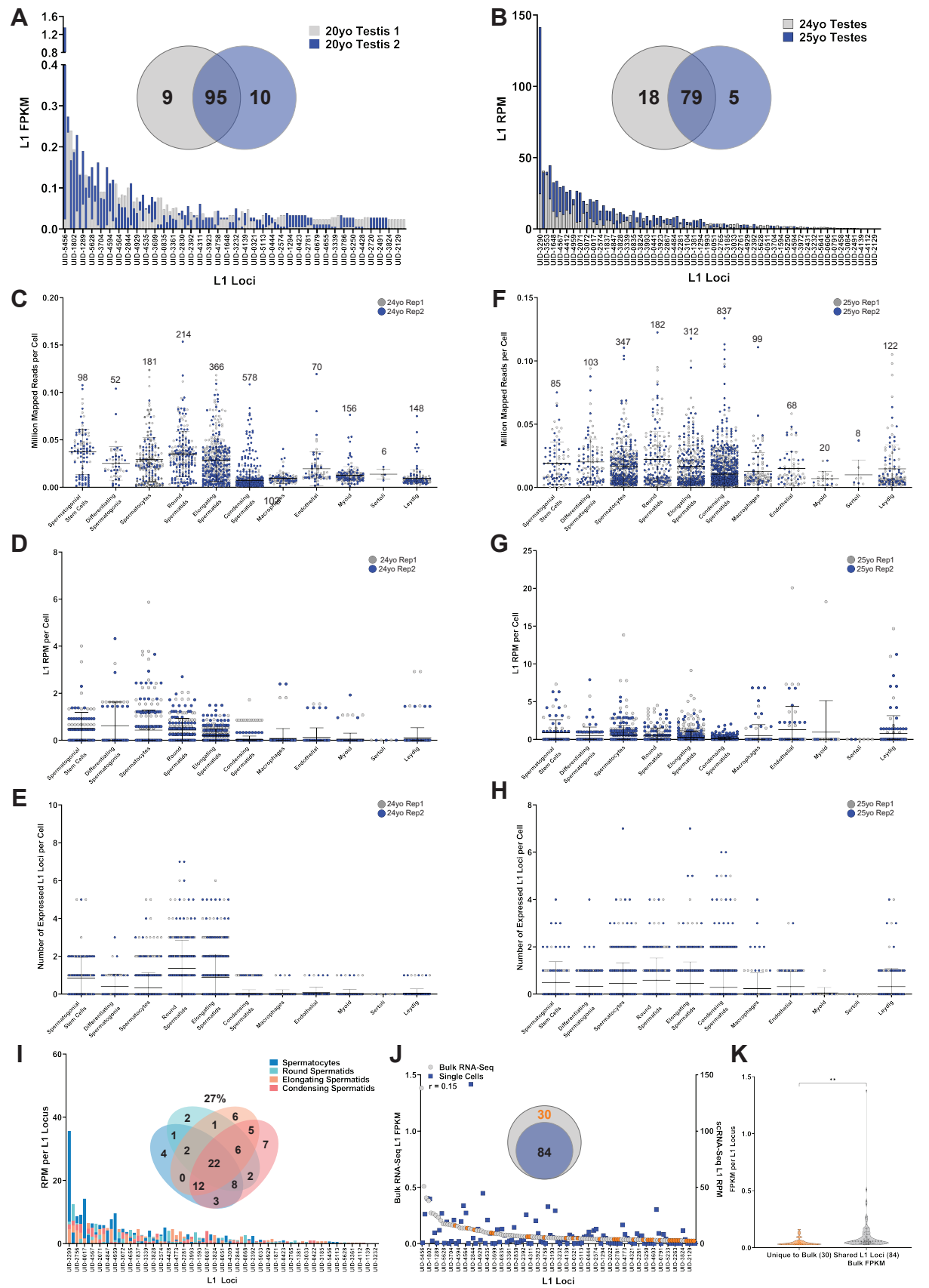

Supplement: Supplementary file 9 — Additional file 9. Analysis of L1 mRNA expression in human testes with bulk RNA-Seq and scRNA-Seq. A. The L1 FPKM values for L1 loci listed on the X-axis are shown for two 20 yo human testis bulk RNA-Seq samples. The number of expressed L1 loci shared between the two replicates is shown in the Venn diagram with the percentage of shared expressed loci indicated above the diagram. B. The L1 RPM values for L1 loci listed on the X-axis are shown for a 24 yo and a 25 yo human testis scRNA-Seq sample. The number of expressed L1 loci shared between the two replicates is shown in the Venn diagram with the percentage of shared expressed loci indicated above the diagram. C. The million mapped reads per cell for all cell types in the 24 yo scRNA-Seq dataset is shown in the scatter plot. Statistics are shown for sperm cells and sperm progenitor cell types only. D. The L1 mRNA expression level per cell for all 24 yo testis cell types is shown in the scatter plot. E. The number of expressed L1 loci per cell for all 24 yo testis cell types is shown in the scatter plot. F. The million mapped reads per cell for all cell types in the 24 yo scRNA-Seq dataset is shown in the scatter plot. Statistics are shown for sperm cells and sperm progenitor cell types only. G. The L1 mRNA expression level per cell for all 25 yo testis cell types is shown in the scatter plot. H. The number of expressed L1 loci per cell for all 25 yo testis cell types is shown in the scatter plot. I. The L1 RPM values for L1 loci indicated on the X-axis are shown for each sperm cell type in the 25 yo human testis sample. The Venn Diagram shows the number of expressed L1 loci shared by the sperm cell types with the percentage of shared expressed L1 loci indicated above the diagram. J. The L1 FPKM values for bulk RNA-Seq (left y-axis) and L1 RPM values for scRNA-Seq (right y-axis) of human testis are shown in the dot-plot. Orange circles indicate L1 loci with detected expression in the bulk dataset that were not detec [file 13100_2022_276_MOESM9_ESM.pdf]

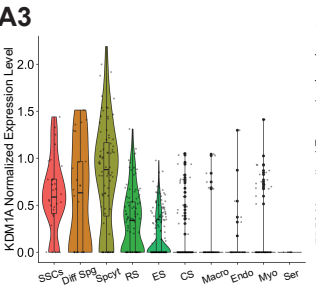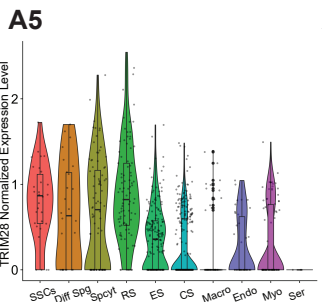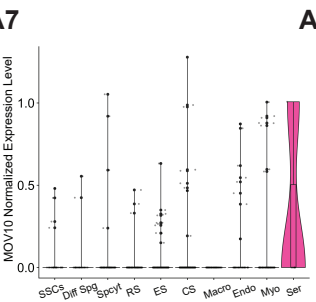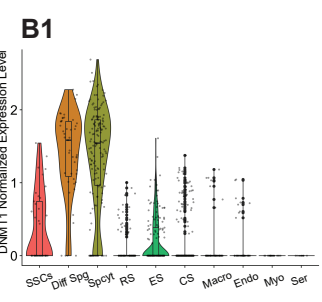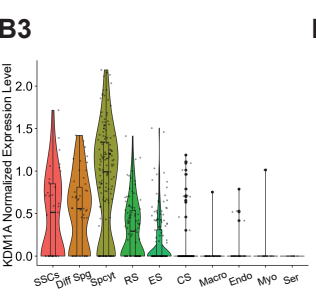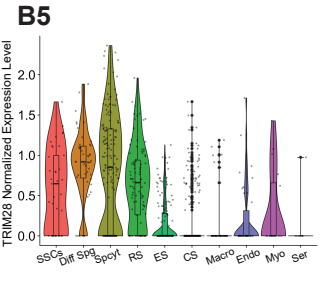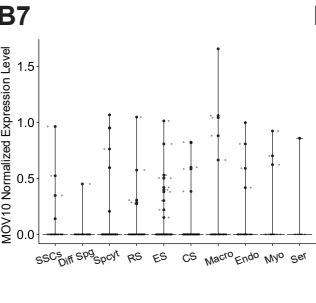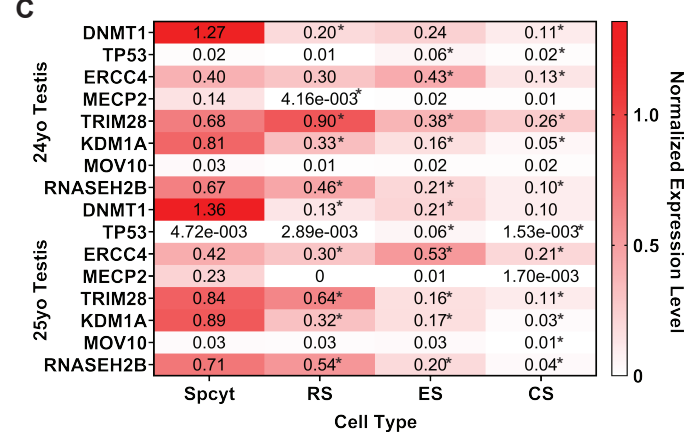

Supplement: Supplementary file 10 — Additional file 10. Expression patterns of genes involved in limiting L1 expression and integration in humans. Cell types are abbreviated as follows: Spermatogonial Stem Cells (SSCs), Differentiating Spermatogonia (Diff Spg), Spermatocytes (Spcyt), Round Spermatids (RS), Elongating Spermatids (ES), Condensing Spermatids (CS), Macrophages (Macro), Endothelial (Endo), Myoid (Myo), and Sertoli (Ser). A. 24 yo Human Testis gene expression patterns for DNMT1 (A1), MECP2 (A2), KDM1A (A3), TP53 (A4), TRIM28 (A5), ERCC4 (A6), MOV10 (A7), and RNASEH2B (A8). B. 25 yo Human Testis gene expression patterns for DNMT1 (B1), MECP2 (B2), KDM1A (B3), TP53 (B4), TRIM28 (B5), ERCC4 (B6), MOV10 (B7), and RNASEH2B (B8). C. The heat map shows the normalized expression patterns for each cell type and gene in 24 yo Testis (top) and 25 yo Testis (bottom). The asterisks indicate a significant change in gene expression in the cell type compared to the preceding cell type in the row (P < 0.05). [file 13100_2022_276_MOESM10_ESM.pdf]
