# Supplementary material for: SCIFER: approach for analysis of LINE-1 mRNA expression in single cells at a single locus resolution
Source: Mob DNA. 2022 Aug 26;13:21. doi: 10.1186/s13100-022-00276-0 (PMC9413895; doi:10.1186/s13100-022-00276-0)
Supplement: Supplementary file 11 — Additional file 11. Directions for running Single Cell Implementation to Find Expressed Retrotransposons (SCIFER). [file 13100_2022_276_MOESM11_ESM.docx]

**Steps for Single Cell RNA-Seq Analysis**

This pipeline is for use with single-cell RNA sequencing reads produced using 10X Genomics technologies. Reads should be processed using Cell Ranger software before beginning. **Cellranger mkfastq** will produce the required fastq files (R1, R2, I1) and **cellranger count** will assign barcodes and clusters for fastq input files.

**1. Reassign barcode-UMIs in R1 fastq file to header in R2 fastq file.**

The single cell barcode-UMIs are in R1. **Print every other line to obtain only barcode-UMIs/indeces and scores. Delete even lines to obtain only barcode-UMIs.**

$ awk '!(NR%2)' [FILE]_S1_L001_R1_001.fastq > [FILE]_S1_L001_R1_001barcodescore.fastq

$ sed 'n; d' [FILE]_S1_L001_R1_001barcodescore.fastq > [FILE]_S1_L001_R1_001barcode.fastq

**Print first 26 letters of reads in R1.fastq to obtain barcodes and UMIs. Then print first 16 letters of reads in R1.fastq to obtain barcodes.**

$ cut -c-28 [FILE]_S1_L001_R1_001barcode.fastq > [FILE]_S1_L001_R1_001barcode_28char.fq

$ cut -c-16 [FILE]_S1_L001_R1_001barcode.fastq > [FILE]_S1_L001_R1_001barcode_16char.fq

**Add delimiter to end of barcodes and combine with list of barcode-UMIs.**

$ sed 's/$/\./' [FILE]_S1_L001_R1_001barcode_16char.fq > [FILE]_S1_L001_R1_001barcode_16char_period.fq

$ paste -d'\0' [FILE]_S1_L001_R1_001barcode_16char_period.fq

[FILE]_S1_L001_R1_001barcode_28char.fq > ${prefix}_1_barcode_28char_16char.fq

**Print headers from [FILE]_S1_L001_R2_001.fastq file.**

$ grep -w “@SRR6129051” [FILE]_S1_L001_R2_001.fastq > [FILE]_S1_L001_R2_001_hdr.fastq

**Substitute job name ‘CAV30ANXX’ with barcode. Change as needed for sequence header.**


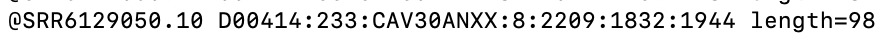


**Delete everything after D00414:.**

$ sed 's/233:.*//g' [FILE]_S1_L001_R2_001_hdr.fastq > [FILE]_S1_L001_R2_001_hdr1.fastq

**Paste together file containing the beginning half of header and the file containing barcode-UMIs together.**

$ paste -d'\0' [FILE]_S1_L001_R2_001_hdr1.fastq [FILE]_S1_L001_R1_001_ barcode_28char_16char.fastq > [FILE]_S1_L001_R2_001_hdr1_barcode.fastq


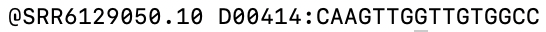


**Delete headers from original [FILE]_S1_L001_R2_001.fastq files**

$ sed '/@SRR6129051/d' [FILE]_S1_L001_R2_001.fastq > [FILE]_S1_L001_R2_001_no header.fastq

**Space file with headers to be compatible with file with reads.**

**Add three spaces between lines.**

$ sed 'G;G;G' [FILE]_S1_L001_R2_001_hdr1_barcode.fastq > [FILE]_S1_L001_R2_001_hdr1_barcode_spaced.fastq

**Space file with reads and no headers to be compatible with file that contains barcode-UMI embedded headers**

**Add a blank row every three rows**

$ sed '0~3 a\\' [FILE]_S1_L001_R2_001_no_header.fastq > [FILE]_S1_L001_R2_001_noheader_spaced.fastq

**Add one blank row at top**

$ sed '1i\\' [FILE]_S1_L001_R2_001_noheader_spaced.fastq > [FILE]_S1_L001_R2_001_no header_spaced_topline.fastq

**Paste together file with barcoded headers and file containing reads**

$ paste -d'\0' [FILE]_S1_L001_R2_001_hdr1_barcode_spaced.fastq [FILE]_S1_L001_R2_001_noheader_spaced_topline.fastq > [FILE]_reads_barcodes.fastq

**Delete all spaces in .fastq file to prevent deletion of barcode after alignment**

$ sed 's/ //g' [FILE]_reads_barcodes.fastq > [FILE]_reads_barcodes_nospace.fastq

**2. Align all reads using bowtie and stringent, unique alignment parameters.**

$ /lustre/project/vperepe/apps/bowtie-0.12.8/bowtie -p 10 -m 1 -S -y -v 3 --chunkmbs 8184 /lustre/project/vperepe/apps/bowtieIndexes/mm10 [FILE]_reads_barcodes_nospace.fastq | samtools view -hbuS - | samtools sort -o [FILE]_reads_barcodes_bowtie_mm10_sorted.bam

**Remove duplicate reads**

$ samtools rmdup [FILE]_reads_barcodes_bowtie_mm10_sorted.bam > [FILE]_reads_barcodes_bowtie_mm10_sorted_rmdup.bam

**3. Extract aligned reads and extract cell-specific alignments based on barcode. Add a colon before each barcode in list_of_barcodes.txt file to ensure the extraction is based on barcode matches alone (instead of reads that potentially contain barcode in sequence).**

$ samtools view -h [FILE]_reads_barcodes_bowtie_mm10_sorted_rmdup.bam | awk 'substr($0,1,1) == "@" || $2 == 0 {print}' | samtools view -bS - > [FILE]_reads_barcodes_bowtie_mm10_sorted_rmdup_top.bam

$ samtools view -h [FILE]_reads_barcodes_bowtie_mm10_sorted_rmdup.bam | awk 'substr($0,1,1) == "@" || $2 == 16 {print}' | samtools view -bS - > [FILE]_reads_barcodes_bowtie_mm10_sorted_rmdup_bottom.bam

$ samtools merge [FILE]_reads_barcodes_bowtie_mm10_sorted_rmdup_top_bottom.bam [FILE]_reads_barcodes_bowtie_mm10_sorted_rmdup_top.bam [FILE]_reads_barcodes_bowtie_mm10_sorted_rmdup_bottom.bam

$ samtools view -h [FILE]_reads_barcodes_bowtie_mm10_sorted_rmdup_top_bottom.bam > [FILE]_reads_barcodes_bowtie_mm10_sorted_rmdup_top_bottom.sam

$ while read line in file; do grep $line [FILE]_reads_barcodes_bowtie_mm10_sorted_rmdup_top_bottom.sam > ${line}_barcode.sam; done < list_of_barcodes.txt

**##These files will be used to determine number of mapped reads using:**

$ for i in ls *.bam; do echo $(cat ${i} | samtools view -F 0x904 -c)_$i; done

**4. Extract reads that align to expressed L1 loci and count duplicate barcode-UMIs.**

$ samtools view -b -h -L expressed_L1_bedfile_plus.bed [FILE]_reads_barcodes_bowtie_mm10_sorted_rmdup_top.bam > [FILE]_reads_barcodes_bowtie_mm10_sorted_rmdup_top_expressedL1.bam

$ samtools view -b -h -L expressed_L1_bedfile_minus.bed [FILE]_reads_barcodes_bowtie_mm10_sorted_rmdup_bottom.bam > [FILE]_reads_barcodes_bowtie_mm10_sorted_rmdup_bottom_expressedL1.bam

$ samtools merge [FILE]_reads_barcodes_bowtie_mm10_sorted_rmdup_top_bottom_expressedL1.bam [FILE]_reads_barcodes_bowtie_mm10_sorted_rmdup_top_expressedL1.bam [FILE]_reads_barcodes_bowtie_mm10_sorted_rmdup_bottom_expressedL1.bam

$ samtools view [FILE]_reads_barcodes_bowtie_mm10_sorted_rmdup_bottom_expressedL1.bam > [FILE]_reads_barcodes_bowtie_mm10_sorted_rmdup_bottom_expressedL1.sam

**Cut beginning of header that contains barcode and barcode-UMI. Change as needed for sequence header.**

$ cut -c-51 [FILE]_reads_barcodes_bowtie_mm10_sorted_rmdup_bottom_expressedL1.sam > [FILE]_reads_barcodes_bowtie_mm10_sorted_rmdup_bottom_expressedL1_51.txt

**Count duplicated barcode-UMIs.**

$ sort [FILE]_reads_barcodes_bowtie_mm10_sorted_rmdup_bottom_expressedL1_51.txt |uniq -c| sort -n -r > [FILE]_reads_barcodes_bowtie_mm10_sorted_rmdup_bottom_expressedL1_51_count.txt

$ awk '{

for ( i=1; i<=NF; i++ )

dict[$i]++;

}

END{

for (key in dict)

if(dict[key]>1)

print dict[key] " : " key

}' [FILE]_reads_barcodes_bowtie_mm10_sorted_rmdup_bottom_expressedL1_51_count.txt

**5. Create a list of unique barcode UMIs and duplicated barcode UMIs. Extract reads from each list and only keep one of each duplicated barcode-UMI.**

$ grep -w "1" [FILE]_reads_barcodes_bowtie_mm10_sorted_rmdup_bottom_expressedL1_51_count.txt > [FILE]_reads_barcodes_bowtie_mm10_sorted_rmdup_bottom_expressedL1_51_count_unique.txt

$ grep -v -w "1" [FILE]_reads_barcodes_bowtie_mm10_sorted_rmdup_bottom_expressedL1_51_count.txt > [FILE]_reads_barcodes_bowtie_mm10_sorted_rmdup_bottom_expressedL1_51_count_nonunique.txt

$ while read line in file; do grep $line [FILE]_reads_barcodes_bowtie_mm10_sorted_rmdup_bottom_expressedL1.sam > ${line}_unique.sam; done < [FILE]_reads_barcodes_bowtie_mm10_sorted_rmdup_bottom_expressedL1_51_count_unique.txt

$ while read line in file; do grep $line [FILE]_reads_barcodes_bowtie_mm10_sorted_rmdup_bottom_expressedL1.sam | head -1 > ${line}_nonunique.sam; done < [FILE]_reads_barcodes_bowtie_mm10_sorted_rmdup_bottom_expressedL1_51_count_nonunique.txt

**6. Add header to all output .sam files, convert to .bam, and merge all files.**

$ for file in *.sam; do (head -30 top30_sam_hg38_MCF7.txt ; cat $file) > ${file}_header.sam; done

$ for file in *_header.sam; do samtools view -bS $file> ${file}.bam; done

$ samtools merge [FILE]_L1exp_reads_unique.bam *._header.bam

$ samtools view [FILE]_L1exp_reads_unique.bam > [FILE]_L1exp_reads_unique.sam

**7. Extract cell barcodes into individual .sam files, convert to bam, strand separate reads and count reads that overlap with repeat element annotations.**

$ while read line in file; do grep $line [FILE]_L1exp_reads_unique.bam

| head -1 > ${line}_barcode.sam; done < list_of_cell_barcodes.txt

$ for file in *.sam; do (head -30 top30_sam_hg38_MCF7.txt ; cat $file) > ${file}_header.sam; done

$ for file in *_header.sam; do samtools view -bS $file> ${file}.bam; done

**8. Strand separate reads and count reads that overlap with repeat element annotations.**

$ samtools view -h ${file}.bam | awk 'substr($0,1,1) == "@" || $2 == 0 {print}' | samtools view -bS - > ${file}_top.bam

$ samtools view -h ${file}.bam | awk 'substr($0,1,1) == "@" || $2 == 16 {print}' | samtools view -bS - > ${file}_bottom.bam

$ bedtools coverage -abam expressed_L1_bedfile_plus.bed -b ${file}_top.bam > [FILE]_top_plus.txt

$ bedtools coverage -abam expressed_L1_bedfile_minus.bed -b ${file}_bottom.bam > [FILE]_bottom_minus.txt

**9. Analysis of read counts**

**Combine read counts from all barcode-specific coverage.txt files for each cluster**

$ cut -f 10 *plus.txt >> all_read_counts_plus.txt

$ cut -f 10 *minus.txt >> all_read_counts_minus.txt

**Sort combined file into individual columns. 305 should be changed according to the number of repeat element coordinates in the reference list. The output file contains reads per repeat element locus with reads corresponding to each barcode in separate columns. Columns are delimited with a comma for ease of import into excel for further analysis.**

$ awk '{a[NR%305] = a[NR%305] (NR<=305 ? "" : ",") $0}

END{for (i = 1; i <= 305; i++) print a[i%305]}' < all_read_counts_plus.txt > all_plus.csv

$ awk '{a[NR%305] = a[NR%305] (NR<=305 ? "" : ",") $0}

END{for (i = 1; i <= 305; i++) print a[i%305]}' < all_read_counts_minus.txt > all_minus.csv

**10. Calculate FPKM using read output files (step 9) and number of million mapped reads (step 3). Change the 2483 number to the number of expressed L1 loci per file used in step 8. Change the number 6 according to gene length. 6 is for the length of L1 (6kb). Perform the following in Python:**

$ import glob

$ import pandas as pd

$ reads=glob.glob('*_plus.csv')

$ for f in reads:

millmap=f.replace("_plus.csv","_millmap.csv")

df_1=pd.read_csv(f,delimiter=',',header=None)

df_2=pd.read_csv(millmap,delimiter=',',header=None)

df_expand=pd.concat([df_2]*305, ignore_index=True)

mappedreads6=df_expand*6

FPKM=df_1/mappedreads6

j=f+"_FPKM.csv"

FPKM.to_csv(j, header=None)

$ reads=glob.glob('*_minus.csv')

$ for f in reads:

millmap=f.replace("_minus.csv","_millmap.csv")

df_1=pd.read_csv(f,delimiter=',',header=None)

df_2=pd.read_csv(millmap,delimiter=',',header=None)

df_expand=pd.concat([df_2]*305, ignore_index=True)

mappedreads6=df_expand*6

FPKM=df_1/mappedreads6

j=f+"_FPKM.csv"

FPKM.to_csv(j, header=None)
